# Intranasally administered extracellular vesicles from human induced pluripotent stem cell-derived neural stem cells quickly incorporate into neurons and microglia in 5xFAD mice

**DOI:** 10.3389/fnagi.2023.1200445

**Published:** 2023-06-22

**Authors:** Sahithi Attaluri, Jenny Jaimes Gonzalez, Maha Kirmani, Andrew D. Vogel, Raghavendra Upadhya, Maheedhar Kodali, Leelavathi N. Madhu, Shama Rao, Bing Shuai, Roshni S. Babu, Charles Huard, Ashok K. Shetty

**Affiliations:** Institute for Regenerative Medicine, Department of Cell Biology and Genetics, School of Medicine, Texas A&M University Health Science Center, College Station, Texas, United States

**Keywords:** Alzheimer’s disease, astrocytes, extracellular vesicles, human neural stem cells, human pluripotent stem cells, interstitial space, intranasal administration, microglia

## Abstract

**Introduction:**

Extracellular vesicles (EVs) released by human-induced pluripotent stem cell (hiPSC)-derived neural stem cells (NSCs) have robust antiinflammatory and neurogenic properties due to therapeutic miRNAs and proteins in their cargo. Hence, hiPSC-NSC-EVs are potentially an excellent biologic for treating neurodegenerative disorders, including Alzheimer’s disease (AD).

**Methods:**

This study investigated whether intranasally (IN) administered hiPSC-NSC-EVs would quickly target various neural cell types in the forebrain, midbrain, and hindbrain regions of 3-month-old 5xFAD mice, a model of β-amyloidosis and familial AD. We administered a single dose of 25 × 10^9^ hiPSC-NSC-EVs labeled with PKH26, and different cohorts of naïve and 5xFAD mice receiving EVs were euthanized at 45 min or 6 h post-administration.

**Results:**

At 45 min post-administration, EVs were found in virtually all subregions of the forebrain, midbrain, and hindbrain of naïve and 5xFAD mice, with predominant targeting and internalization into neurons, interneurons, and microglia, including plaque-associated microglia in 5xFAD mice. EVs also came in contact with the plasma membranes of astrocytic processes and the soma of oligodendrocytes in white matter regions. Evaluation of CD63/CD81 expression with the neuronal marker confirmed that PKH26 + particles found within neurons were IN administered hiPSC-NSC-EVs. At 6 h post-administration, EVs persisted in all cell types in both groups, with the distribution mostly matching what was observed at 45 min post-administration. Area fraction (AF) analysis revealed that, in both naïve and 5xFAD mice, higher fractions of EVs incorporate into forebrain regions at both time points. However, at 45 min post-IN administration, AFs of EVs within cell layers in forebrain regions and within microglia in midbrain and hindbrain regions were lower in 5xFAD mice than naïve mice, implying that amyloidosis reduces EV penetrance.

**Discussion:**

Collectively, the results provide novel evidence that IN administration of therapeutic hiPSC-NSC-EVs is an efficient avenue for directing such EVs into neurons and glia in all brain regions in the early stage of amyloidosis. As pathological changes in AD are observed in multiple brain areas, the ability to deliver therapeutic EVs into various neural cells in virtually every brain region in the early stage of amyloidosis is attractive for promoting neuroprotective and antiinflammatory effects.

## 1. Introduction

Transplantation of neural stem cells (NSCs) can replace some of the lost neurons, promote synapse growth, repair the disrupted neural circuitry and improve brain function in animal models of Alzheimer’s disease (AD) (Blurton-Jones et al., 2009; Fujiwara et al., 2013; Ager et al., 2015; Li et al., 2016; McGinley et al., 2018; Zhang et al., 2019; Lu et al., 2021; Campos et al., 2022; Yue et al., 2022). However, one study has demonstrated no cognitive benefits of human NSC grafting in a model of AD (Marsh et al., 2017). Moreover, AD pathogenesis involves multiple brain regions involving degeneration of different subtypes of neurons (Frozza et al., 2018; Wang et al., 2020; Veitch et al., 2022), which will likely require region-specific cell therapy for appropriate brain repair and functional recovery, but NSCs have limited ability to differentiate into region-specific neuronal phenotypes after transplantation (Fricker et al., 1999). While direct differentiation of human embryonic stem cells (hESCs) or human induced pluripotent stem cells (hiPSCs) into specific neuronal phenotypes can provide the required donor neurons such as glutamatergic, cholinergic, and GABA-ergic neurons for grafting (Liu et al., 2013; Hu et al., 2016; Upadhya et al., 2019; Fitzgerald et al., 2020; Muñoz et al., 2020), it would be difficult to identify specific regions of the AD brain that display loss of such neurons during disease progression. Furthermore, such a multi-brain region grafting approach is highly invasive. Other concerns about the grafting of hPSC-derived NSCs or neural cells exist. For example, allografting of neural cells requires long-term or lifelong immunosuppression to maintain the survival of graft-derived neurons, which may induce multiple adverse effects (Katabathina et al., 2016; Levi et al., 2019). While a patient-specific cell therapy approach [i.e., grafting of neural cells derived from human-induced PSCs (hiPSCs)] could circumvent the above issues, including ethical considerations, such an approach is likely expensive and might not be feasible for treating a large number of patients (Kiskinis and Eggan, 2010; Han et al., 2020). Moreover, the translation of patient-specific hiPSC-derived cell therapy to the clinic has been hampered by safety issues, which include the possible genetic instability leading to incomplete differentiation or teratoma formation (Bedel et al., 2017; Itakura et al., 2017; Martin et al., 2020).

Considering the concerns mentioned above, the use of nano-sized extracellular vesicles (EVs) derived from NSCs or other neural cell types differentiated from hPSCs as a therapeutic biologic is attractive (Vogel et al., 2018; Liu et al., 2020; Spellicy et al., 2020; Upadhya et al., 2020a,b; Tian et al., 2021). The use of EVs can also be justified by reports suggesting that the therapeutic outcomes of stem cells in various models of human diseases are primarily due to the paracrine actions of their secretome (Drago et al., 2013; Willis et al., 2020a; Santamaria et al., 2021). EVs are the principal components of the secretome because of their ability to pass on proteins, nucleic acids, and lipids to injured cells in the host (Long et al., 2017; Vogel et al., 2018; Kodali et al., 2020, 2023). Thus, because of their proficiency to mimic the beneficial effects of parental cells, stem cell-derived EVs have attracted immense attention as therapeutic alternatives to stem cells in treating neurological and neurodegenerative diseases (Upadhya and Shetty, 2019, 2021a,2021b; Upadhya et al., 2020a,b; Shetty and Upadhya, 2021). Studies have already reported the potential of stem cell-derived EVs in treating traumatic brain injury (Kim et al., 2016; Sun et al., 2020; Bambakidis et al., 2022; Kodali et al., 2023), Parkinson’s disease (Chen et al., 2020; Upadhya and Shetty, 2021b; Lee et al., 2022), stroke (Kuang et al., 2020; Cai et al., 2021; Tian et al., 2021; Xia et al., 2021), and Alzheimer’s disease (Losurdo et al., 2020; Cone et al., 2021). Furthermore, as required, repeated, non-invasive administrations are feasible with EV therapy (Vogel et al., 2018; Shetty and Upadhya, 2021).

Because the therapeutic effects of exogenously administered stem cell-derived EVs in the central nervous system (CNS) are dependent on the transfer of EV cargo (proteins, nucleic acids, and lipids) into target neurons and glia, an in-depth analysis of their cargo, biological properties, and ability to target different neural cell types in various brain regions after peripheral administration is critical for promoting their use in treating neurodegenerative disorders (Abels and Breakefield, 2016; Vogel et al., 2018; Hill, 2019; Upadhya et al., 2020a). Our previous study has described protocols for generating and isolating therapeutic EVs from hiPSC-NSCs using a combination of anion-exchange chromatography (AEC) and size exclusion chromatography (SEC). Also, by employing several *in vitro* and *in vivo* assays, the study demonstrated that hiPSC-NSC-EVs have robust antiinflammatory and neurogenic effects, suggesting that such EVs could be employed as a biologic to improve brain function in neurodegenerative conditions, including AD (Upadhya et al., 2020b). Furthermore, small RNA sequencing and proteomic studies have also identified the highly enriched miRNA and protein cargo within these EVs (Upadhya et al., 2020a). Moreover, miRNA/protein knockdown studies in a cell culture model have confirmed the role of specific miRNAs/proteins in mediating robust antiinflammatory effects (Upadhya et al., 2022).

However, it is yet to be determined whether hiPSC-NSC-EVs can target different neural cell types in the entire AD brain following intranasal (IN) administration. Such investigation is essential, as pathological changes in AD are observed in both neurons and glia in multiple brain areas, and targeting EVs into different neural cell types may be necessary to induce beneficial transcriptomic changes. Therefore, in this study, we investigated whether a single intranasal (IN) dose (25 × 10^9^) of hiPSC-NSC-EVs labeled with PKH26 would quickly target glutamatergic and GABA-ergic neurons, microglia, astrocytes, and oligodendrocytes in the forebrain, midbrain, and hindbrain regions of 3-month-old male 5xFAD mice, a model of early-onset amyloidosis. Our results demonstrate that within 45 min post-IN administration, PKH26-labeled EVs expressing CD63/CD81 permeated all subregions of the forebrain, midbrain, and hindbrain of 5xFAD mice and targeted different neural cell types with predominant incorporation into neurons and microglia. Notably, at 6 h post-IN administration, EVs persisted in all neural cell types with comparable distribution patterns. Overall, the study demonstrated for the first time that IN administration of hiPSC-NSC-EVs is an efficient avenue for directing such EVs into neurons and glia in all subregions of the forebrain, midbrain, and hindbrain in 5xFAD mice.

## 2. Materials and methods

### 2.1. Animals

Three-month-old male 5xFAD mice, a model of early onset of amyloidosis (one of the pathological hallmarks of AD), were employed in this study. These mice display five human familial AD mutations driven by the mouse Thy1 promoter (Oakley et al., 2006). These mice were bred in-house and kept at Texas A&M University’s vivarium. 5xFAD transgenic male mice and B6SJLF1/J female mice used in the crossing were purchased from Jackson Laboratories (Cat No: 34840-JAX and 100012-JAX). Newborn pups were identified as 5xFAD and wild type through genotyping on postnatal day 15. Animals were maintained in a climate-controlled room with a 12:12-h light-dark cycle and free access to food and water. The Animal Care and Use Committee at Texas A&M University approved all experimental procedures performed in this study.

### 2.2. Study design

Two groups of mice were employed to investigate EV incorporation into different brain regions: a naïve group comprising littermates of 5xFAD mice (*n* = 8) and an AD group comprising 5xFAD mice (*n* = 8). One cohort (*n* = 4/group) of mice was sacrificed 45 min after IN administration, and another cohort of animals (*n* = 4/group) was perfused 6 h after IN administration of EVs. Different steps involved in the experimental design are (1) Isolation of EVs from NSCs. (2) IN administration of EVs to the animals. (3) perfusion of animals at different time points (45min and 6 h) after IN administration of EVs. (4) Analysis of incorporation of EVs into different brain regions.

### 2.3. Isolation characterization and labeling of hiPSC-NSC derived EVs

The protocols for the preparation, selection, characterization, culture, isolation, and labeling of EVs from hiPSC-NSCs are detailed in our previous report (Upadhya et al., 2020a). Briefly, passage-11 hiPSC-NSCs were cultured, and the complete culture medium (CCM) was replaced with a fresh CCM after 2–3 days. Once the cells attained ∼70% confluency, the cells were dislodged with 1 U/ml dispase (Gibco), rinsed with NSC media (Gibco), and plated at 500 cells per cm^2^ into 150 × 20 mm tissue culture plates (Corning) in NSC expansion medium. The media was retrieved and used for isolating EVs or stored at −80°C for further use once the NSCs attained 90% confluency. After passive thawing, EVs were isolated from the spent media using AEC and SEC methods and characterized, as reported in our previous study (Upadhya et al., 2020a). The concentration and size of the EV particles were determined using nanoparticle tracking analysis in Nanosight. Western blots were employed to confirm the expression of EV-specific marker proteins such as CD81 and apoptosis-linked 2 interacting protein X (ALIX) in hiPSC-NSC-EVs. Furthermore, the lack of contamination of EV preparations with deep cellular proteins was investigated through Western blotting for calnexin and cytochrome c. Moreover, the concentration of CD63 per mg of total EV protein was measured using ELISA (R&D systems). NSC-EV size and morphology were also examined through transmission electron microscopy (TEM) (Upadhya et al., 2020a). According to the manufacturer’s instructions, the EVs were labeled with PKH26 (Sigma), and the free dye was separated from the bound dye by ultrafiltration using 10 kDa MWCO (Molecular Weight Cut-off) filter columns (Sartorius, Gottingen, Germany). Nanosight analysis was performed again to determine the size and concentration of PKH26-labeled EVs. To ensure that our labeled hiPSC-NSC-EVs were devoid of free dye or aggregates, we performed a cell culture study, where hiPSC-derived NSCs were incubated for an hour with PKH26 labeled hiPSC-NSC-EVs (1 × 10^9^) or PKH26 dye solution that was subjected to similar incubation and filtration procedures as the PKH26-labeled EVs. We investigated PKH26 + structures (red particles) in neuronal stem cell cultures incubated with PKH26-labeled EVs or PKH26 dye solution undergoing the identical incubation and filtration steps as the PKH26-labeled EVs to validate that the procedure employed in the study resulted in a suspension of PKH26-labeled EVs containing minimal or no free PKH26 dye.

### 2.4. Intranasal administration of EVs

hiPSC-NSC-EVs were administered intranasally to both naive and 5xFAD mice. IN administration utilized both nostrils. The nostrils of each animal were treated with 20 μL of hyaluronidase (100 U; H3506; Sigma-Aldrich) in sterile PBS solution to increase the permeability of the nasal mucous membrane. Thirty min later, each mouse was administered with 25 × 10^9^ labeled EVs, as detailed in our previous report (Long et al., 2017; Kodali et al., 2019).

### 2.5. Tissue processing and immunofluorescence studies

Subgroups of naive and 5xFAD mice were intracardially perfused 45 min or 6 h after IN administration of EVs using 4% paraformaldehyde. The brains were dissected, fixed overnight in 4% paraformaldehyde, and sectioned using a cryostat (Long et al., 2017; Kodali et al., 2023). Coronal slices of 30-micrometer thickness were cut through the entire brain and serially collected in 24-well plates containing the phosphate buffer. Several sets of serial sections through the entire brain were selected and processed for immunofluorescence procedures, using appropriate primary antibodies against the neuronal and glial markers, as described in our previous reports (Kodali et al., 2019, 2023; Upadhya et al., 2020b). The primary antibodies employed in this study are listed in Table 1. Matching secondary antibodies conjugated to fluorescent probes purchased from either Jackson ImmunoResearch or Thermo Fisher Scientific were employed. Sections were mounted using an antifade-slow fade reagent (Thermo Fisher Scientific). Analysis of PKH26 + labeled EVs with markers of neurons, interneurons, microglia, astrocytes, or oligodendrocytes involved single immunofluorescence protocols, whereas localization of CD63/CD81 in neurons involved dual immunofluorescence methods.

**TABLE 1:** Details of primary antibodies employed in the study.

Primary antibodies	Source	Catalog number	Species reactivity	Dilution
Anti-amyloid beta	Invitrogen	71-5800	Human	1:500
Anti- apoptosis-linked 2 interacting protein X (ALIX)	SantaCruz	Sc-53538	Human, Mouse, Rat	1:1000
Anti-calbindin	Millipore	ABN2192	Mouse, Rat, Human	1:500
Anti-calnexin	ThermoFisher	PA5-34754	Mouse, Rat, Human	1:1000
Anti-cytochrome C	ThermoFisher	10993-1-AP	Mouse, Rat, Human	1:1000
Anti-CD63	BD Pharmingen	556019	Human, Rhesus	1:500
Anti-CD81	BD Pharmingen	555675	Human, Rhesus, Rabbit	1:500
Anti-glial fibrillary acidic protein (GFAP)	Agilent Technologies	GA52461-2		1:3000
Anti-ionized calcium binding adaptor molecule (anti-IBA-1)	Abcam	ab5076	Rat, Human	1:1000
Anti-microtubule-associated protein 2 (MAP-2)	Abcam	ab32454	Mouse, Rat, Human	1:1000
Anti-neuropeptide Y (NPY)	Millipore	AB9608	Rat, Human	1:1000
Anti-neuron-specific nuclear antigen (NeuN)	Millipore	ABN78	Mouse, Rat, Human	1:1000
Anti-parvalbumin (PV)	Millipore	MABN1191	Mouse, Rat, Human	1:1000
Anti-2′,3′-cyclic-nucleotide 3′-phosphodiesterase (anti-CNPase)	Millipore	MAB326	Mouse, Rat, Human	1:500

### 2.6. Confocal microscopy

Optical Z-stacks were sampled from different brain regions using a Nikon confocal microscope. For every marker, the quantitative analysis comprised images from three fields in every sampled subregion of the forebrain, midbrain, and hindbrain. EV incorporation into neurons, interneurons, and microglia in both groups (*n* = 4/group) was analyzed quantitatively. Association of EVs with astrocytes and oligodendrocytes were also examined rigorously as EV incorporation into the soma of these cells were rarely seen. Different regions of the forebrain, including the olfactory bulb (OB), medial prefrontal cortex (mPFC), the somatosensory cortex (SSC), striatum, different subfields of the hippocampus [dentate hilus (DH), dentate granule cell layer (DGCL), CA1, and CA3 subfields], thalamus (TH), hypothalamus (HTH), entorhinal cortex (ECX), and amygdala were analyzed. The Midbrain (MB) and different regions of the hindbrain, including the pons, medulla oblongata (MO), Purkinje cell layer (PCL), and granule cell layer (GCL) of the cerebellum (CBM), were also evaluated. We used a 63X objective lens, a pinhole size of 90 μm and emission wavelengths of 496 and 567 nanometers for green and red fluorescence, respectively. Imaging was performed at 1/24 frames per second.

The following quantifications were performed for naive and 5xFAD mice using Z-section images, the NIS elements image browser, and Image J. (1) The percentages of neurons, interneurons, and microglia incorporating EVs in various brain regions at 45 min and 6 h post-IN administration. (2) The area fraction (AF) of all EVs and average AFs of EVs within neurons and microglia in various brain regions at 45 min and 6 h post-IN administration. (3) Correlation of brain region-specific incorporation of PKH26 + EVs by neurons and microglia in naive and 5xFAD mice vis-a-vis the concentration of total EVs in measured brain regions. (4) AFs of PKH26 + EVs in plaque-associated microglia (PAM) and non-plaque-associated microglia (NPAM). Three sections through every brain region separated by a 450-micrometer distance were imaged in each animal (*n* = 4/group). Three randomly selected Z-sections were used per the chosen brain region in every animal for different quantifications. Furthermore, we also confirmed the incorporation of EVs into soma and dendrites using MAP-2 immunofluorescence.

### 2.7. Statistical analyses

Prism software was used to compare the results statistically. The percentages of neurons, interneurons, and microglia incorporating EVs in various brain regions at 45 min and 6 h post-IN administration between naïve and 5xFAD mice were compared using an unpaired, two-tailed Student’s *t*-test or Mann-Whitney *U* test. The AF of all EVs and average AFs of EVs within neurons and microglia in various brain regions at 45 min and 6 h post-IN administration and AFs of PKH26 + EVs in PAM and NPAM were compared using two-way ANOVA with Tukey’s *post hoc* tests. Correlation of brain region-specific incorporation of PKH26 + EVs by neurons and microglia in naive and 5xFAD mice vis-a-vis the concentration of total EVs in measured brain regions was performed using a Pearson Correlation test. *p* < 0.05 is considered statistically significant.

## 3. Results

### 3.1. Characterization and PKH26 labeling of hiPSC-NSC-EVs–standardization and technical considerations

**FIGURE 1:**
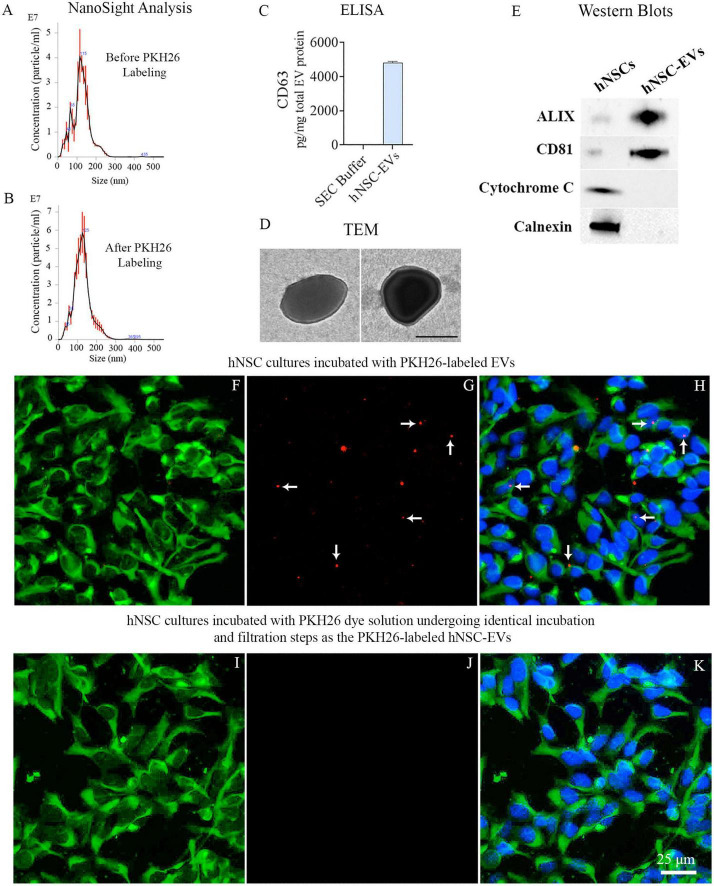
Upper panel: Size and markers of extracellular vesicles (EVs) from human induced pluripotent stem cell derived neural stem cells (hiPSC-NSCs). Graphs **(A,B)** show the size of hiPSC-NSC-EVs before and after PKH26 labeling, measured with a NanoSight. Bar chart **(C)** shows the CD63 concentration in the EV preparation. The image in panel **(D)** displays the morphology and size of hNSC-EVs revealed through transmission electron microscopy. Scale bar, 100 nm. The blots in panel **(E)** demonstrate EV-specific proteins CD81 and ALIX and the absence of the deep cellular proteins cytochrome c and calnexin in hNSC-EVs. Lower panel **(F–K)**: The suspension of PKH26-labeled EVs employed for tracking studies contained minimal or no free PKH26 dye. Human NSC cultures incubated with PKH26-labeled hiPSC-NSC-EVs displayed red particles (i.e., PKH26-labeled EVs), some of which got incorporated into nestin-positive NSCs **(F–H)**. In contrast, no red particles (free PKH26 particles) are evident in human NSC cultures incubated with PKH26 dye solution undergoing the identical incubation and filtration steps as the PKH26-labeled EVs **(I–K)**. Scale bar, 25 μm.

NanoSight analysis of EVs isolated from P11 hiPSC-NSC cultures through AEC and SEC methods and TEM imaging revealed the presence of EVs with a mean size of ∼120 nm (Figure 1 A). NanoSight analysis of EVs before and after PKH26 labeling revealed that the mean size of EVs was 122 nm before PKH26 labeling and 126 nm after PKH26 labeling, implying that the size of EVs does not change significantly after PKH26 labeling (Figures 1A, B). ELISA results confirmed that our hiPSC-NSC-EV preparation contained 4802 pg of CD63 per mg of total EV protein (Figure 1C). TEM analysis confirmed the size and classic morphology of EVs enclosed by double membranes (Figure 1D). Furthermore, Western blots validated that hiPSC-NSC-EVs expressed multiple EV-specific markers such as CD81 and ALIX. Also, the EV lysates lacked the expression of deep cellular proteins such as calnexin and cytochrome C (Figure 1E).

The labeling of hiPSC-NSC-EVs with PKH26 involved the incubation of EVs with PKH26 dye for 20 min at 37°C. The free dye was removed from the bound dye through ultrafiltration using 10 kDa MWCO filter columns. However, to confirm the absence of free dye in the final EV suspension, we prepared a parallel PBS solution mixed with the same concentration of PKH26 dye used for EV labeling with identical incubation and filtration steps as the PKH26-labeled EVs. Next, to determine the presence of free dye, we tested PKH26 labeled EV preparation and PBS preparation on parallel NSC cultures. Red particles were seen in human NSC cultures incubated with PKH26-labeled hiPSC-NSC-EVs (Figures 1F–H). However, such red particles were absent in human NSC cultures treated with PBS preparation undergoing the identical incubation and filtration steps as the PKH26-labeled EVs (Figure 1I–K). Thus, the labeling protocol employed for hiPSC-NSC-EVs is proficient for formulating a suspension of PKH26-labeled EVs comprising minimal or no free PKH26 dye.

### 3.2. IN administered hiPSC-NSC-EVs incorporated into cell bodies of neurons in different regions of forebrain, midbrain and hindbrain in naive and 5xFAD mice

**FIGURE 2:**
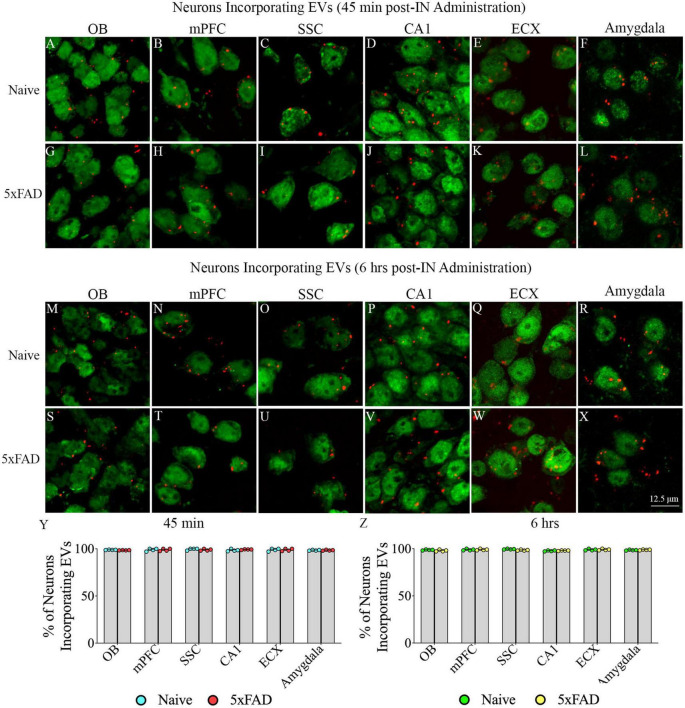
Intranasally administered hiPSC-NSC-EVs incorporate into NeuN + neurons in forebrain regions in naïve and 5xFAD mice. The figure illustrates the incorporation of EVs into NeuN + neurons in different forebrain regions of naïve **(A–F,M–R)** and 5xFAD mice **(G–L,S–X)** at 45 min (*n* = 4/group, **A–L**) and 6 h (*n* = 4/group, **M–X**) post-administration. The bar charts **(Y,Z)** compare the percentages of neurons incorporating EVs in different brain regions between naïve and 5xFAD mice at 45 min **(Y)** and 6 h **(Z)** post-administration. ECX, entorhinal cortex; mPFC, medial prefrontal cortex; OB, olfactory bulb; SSC, somatosensory cortex. Scale bar, 12.5 μm.

**FIGURE 3:**
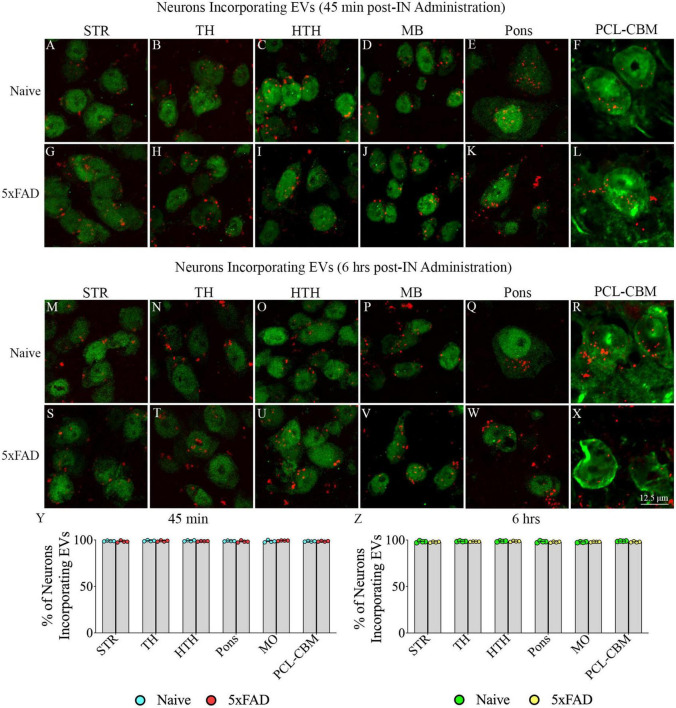
Intranasally administered hiPSC-NSC-EVs incorporate into NeuN + neurons in deeper forebrain regions and the midbrain and hindbrain in naïve and 5xFAD mice. The figure illustrates the incorporation of EVs into NeuN + neurons in different forebrain, midbrain, and hindbrain regions of naïve **(A–F,M–R)** and 5xFAD mice **(G–L,S–X)** at 45 min (*n* = 4/group, **A–L**) and 6 h (*n* = 4/group, **M–X**) post-administration. The bar charts **(Y,Z)** compare the percentages of neurons incorporating EVs in different brain regions between naïve and 5xFAD mice at 45 min **(Y)** and 6 h **(Z)** post-administration. HTH, hypothalamus; MB, midbrain; PCL-CBM, Purkinje cell layer of the cerebellum; STR, striatum; TH, thalamus. Scale bar, 12.5 μm.

Investigation of PKH26 + structures in serial brain tissue sections processed for NeuN immunofluorescence using 0.5 μm thick Z-sections in a confocal microscope revealed widespread incorporation of IN administered hiPSC-NSC-EVs into neurons in the forebrain, midbrain and hindbrain. hiPSC-NSC-EVs incorporated into ∼98% of neurons in both naïve and 5xFAD mice when examined at 45 min and 6 h post-IN administration. Examples of neurons that incorporated PKH26-labeled EVs (red particles) at 45 min and 6 h post-IN administration in different brain regions are illustrated in Figures 2, 3. The illustrated brain regions include the olfactory bulb (Figures 2A, G, M, S), mPFC (Figures 2B, H, N, T), SSC (Figures 1C, I, O, U), CA1 subfield of the hippocampus (Figures 2D, J, P, V), ECX (Figures 2E, K, Q, W), amygdala (Figures 2F, L, R, X), striatum (Figures 3A, G, M, S), TH (Figures 3B, H, N, T), HTH (Figures 3C, I, O, U), MB (Figures 3D, J, P, V), pons (Figures 3E, K, Q, W), and PCL-CBM (Figures 3F, L, R, X). Quantification revealed that at 45 min and 6 h post-IN administration, the percentage of neurons incorporating PKH26-labeled hiPSC-NSC-EVs in different regions of the forebrain, midbrain and hindbrain were comparable between the naïve and 5xFAD groups (Figures 2, 3Y, Z). Thus, IN administration of 25 × 10^9^ hiPSC-NSC-EVs can target a vast majority of neurons in the entire brain of naïve and 5xFAD mice within 45 min.

### 3.3. IN administered hiPSC-NSC-EVs incorporated into cell bodies of interneurons expressing NPY/PV in the forebrain of naive and 5xFAD mice

**FIGURE 4:**
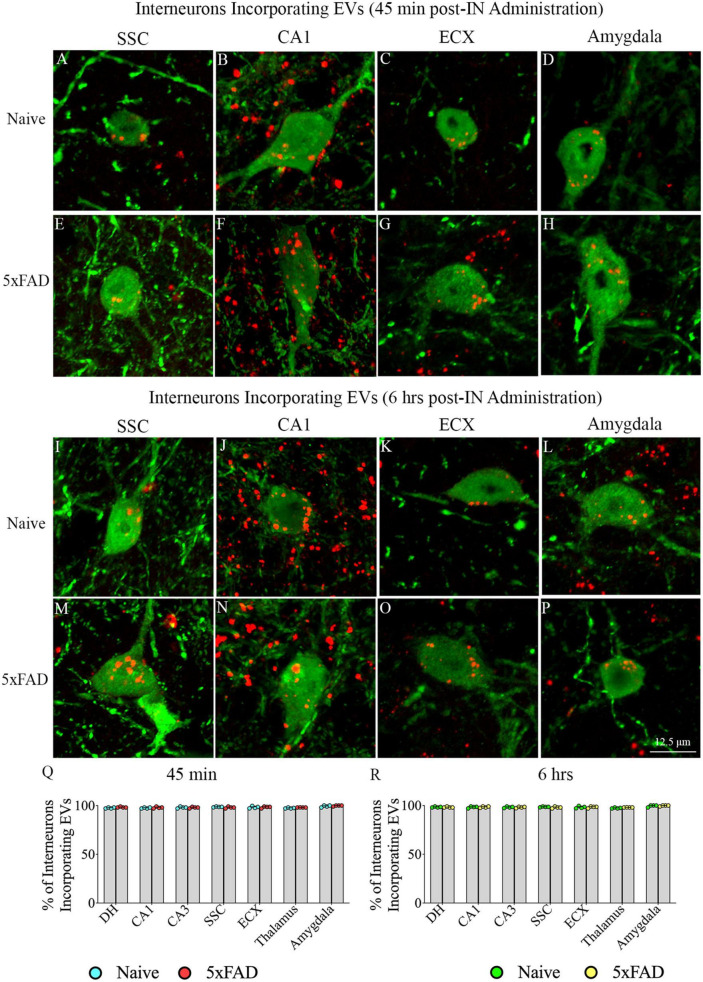
Intranasally administered hiPSC-NSC-EVs incorporate into interneurons. The figure illustrates the incorporation of EVs into NPY/PV-positive interneurons in different forebrain regions of naïve **(A–D,I–L)** and 5xFAD mice **(E–H,M–P)** at 45 min (*n* = 4/group, **A–H**) and 6 h (*n* = 4/group, **I–P**) post-administration. The bar charts **(Q,R)** compare the percentages of NPY/PV-positive interneurons incorporating EVs in different brain regions between naïve and 5xFAD mice at 45 min **(Q)** and 6 h **(R)** post-administration. ECX, entorhinal cortex; SSC, somatosensory cortex. Scale bar, 12.5 μm.

Investigation of PKH26 + structures in serial brain tissue sections processed for NPY/PV immunofluorescence using 0.5 μm thick Z-sections in a confocal microscope revealed incorporation of IN administered hiPSC-NSC-EVs into interneurons in the forebrain. hiPSC-NSC-EVs incorporated into virtually all NPY/PV positive interneurons in both naïve and 5xFAD mice when examined at 45 min and 6 h post-IN administration. Examples of NPY/PV positive interneurons that incorporated PKH26-labeled EVs (red particles) at 45 min and 6 h in the SSC (Figures 4A, E, I, M), CA1 (Figures 4B, F, J, N), ECX (Figures 4C, G, K, O), and amygdala (Figures 4D, H, L, P) are illustrated. Quantification revealed that at both 45 min and 6 h post-IN administration, the percentage of NPY/PV positive interneurons incorporating PKH26-labeled hiPSC-NSC-EVs in different regions of the forebrain were comparable between the naïve and 5xFAD groups (Figures 4Q, R). Thus, IN administration of 25 × 10^9^ hiPSC-NSC-EVs can target a vast majority of NPY/PV positive interneurons in the entire forebrain of naïve and 5xFAD mice within 45 min.

### 3.4. PKH26 + red particles found within neurons expressed EV-specific markers CD63 and CD81

**FIGURE 5:**
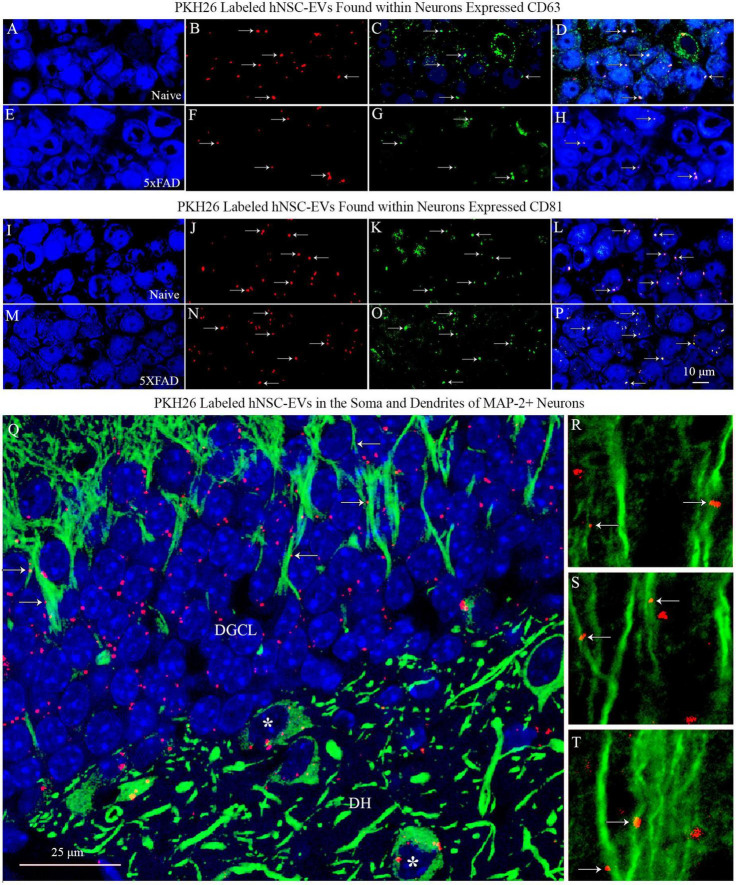
PKH26 labeled hiPSC-NSC-EVs found within neurons expressed EV-specific markers CD63 and CD81 in naïve and 5xFAD mice. The images in panels **(A–H)** demonstrate that PKH26 labeled EVs incorporating into NeuN + neurons in the CA3 subfield of the hippocampus in naïve mice **(A–D)** and 5xFAD mice **(E–H)** co-expressed CD63. Figures **(I–P)** show that PKH26 labeled EVs incorporating into NeuN + neurons in the CA3 subfield of the hippocampus in naïve mice **(I–L)** and 5xFAD mice **(M–P)** co-expressed CD81. Scale bar, 10 μm. Image **(Q)** illustrates the incorporation of IN-administered hNSC-EVs into the soma of MAP-2 + neurons in the dentate hilus (DH, indicated by asterisks) and the dentate granule cell layer (DGCL). Either incorporation or association of PKH26 + EVs with the dendrites of neurons is evident in the DGCL [arrows in panel **(Q)**] and the somatosensory cortex [arrows in panels **(R–T)**]. Scale bar, 25 μm.

To confirm that the PKH26 + red particles found within and outside the soma of neurons in naïve and 5xFAD mice are the IN administered hiPSC-NSC-EVs, we performed careful Z-section analysis of brain tissue sections processed for NeuN and CD63 and NeuN and CD81 dual immunofluorescence. Such analysis revealed that virtually all red particles found within neurons and outside neurons expressed both CD63 and CD81. Thus, PKH26 + red particles found within the brain following IN administration of PKH26-labeled hiPSC-NSC-EVs are indeed EVs and not dye particles (Figures 5A–P). Furthermore, the brain tissue sections processed for MAP-2 immunofluorescence validated the incorporation of IN-administered hiPSC-NSC-EVs into the soma of neurons and the association of hiPSC-NSC-EVs with the dendrites. Figures 5Q–T illustrates examples of EV incorporation into the soma of neurons in the DH and DGCL (Figure 5Q) and EV association with the MAP-2 + dendrites in the DGCL and the SSC (Figures 5Q–T).

### 3.5. IN administered hiPSC-NSC-EVs incorporated into the soma of microglia in different regions of forebrain, midbrain and hindbrain in naive and 5xFAD mice

**FIGURE 6:**
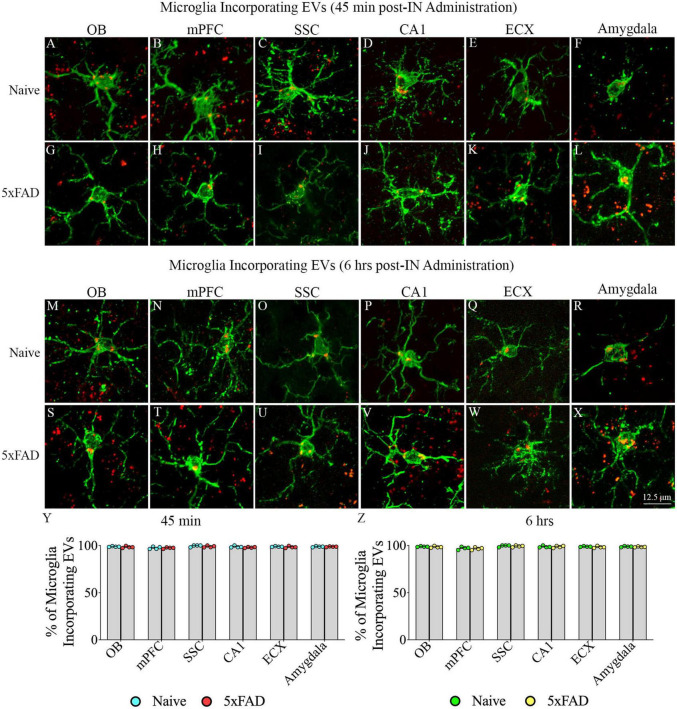
Intranasally administered hiPSC-NSC-EVs incorporate into IBA-1 + microglia in forebrain regions in naïve and 5xFAD mice. The figure illustrates the incorporation of EVs into IBA-1 + microglia in different forebrain regions of naïve **(A–F,M–R)** and 5xFAD mice **(G–L,S–X)** at 45 min (*n* = 4/group, **A–L**) and 6 h (*n* = 4/group, **M–X**) post-administration. The bar charts **(Y,Z)** compare the percentages of microglia incorporating EVs in different brain regions between naïve and 5xFAD mice at 45 min **(Y)** and 6 h **(Z)** post-administration. ECX, entorhinal cortex; mPFC, medial prefrontal cortex; OB, olfactory bulb; SSC, somatosensory cortex. Scale bar, 12.5 μm.

**FIGURE 7:**
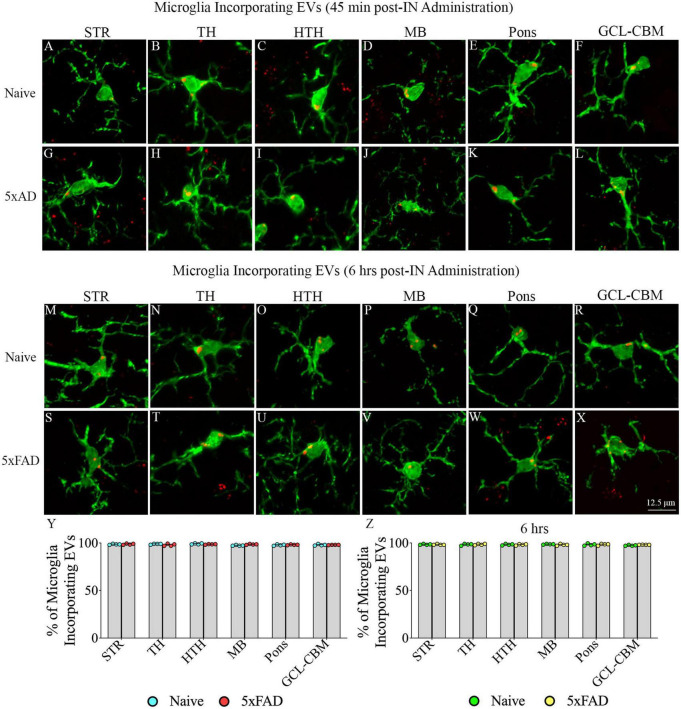
Intranasally administered hiPSC-NSC-EVs incorporate into IBA-1 + microglia in deeper forebrain regions, midbrain and hindbrain in naïve and 5xFAD mice. The figure illustrates the incorporation of EVs into IBA-1 + microglia in different forebrain, midbrain, and hindbrain regions of naïve **(A–F,M–R)** and 5xFAD mice **(G–L,S–X)** at 45 min (*n* = 4/group, **A–L**) and 6 h (*n* = 4/group, **M–X**) post-administration. The bar charts **(Y,Z)** compare the percentages of microglia incorporating EVs in different brain regions between naïve and 5xFAD mice at 45 min **(Y)** and 6 h **(Z)** post-administration. GCL-CBM, granule cell layer of the cerebellum; HTH, hypothalamus; MB, midbrain; STR, striatum; TH, thalamus. Scale bar, 12.5 μm.

Examination of PKH26 + structures in serial brain tissue sections processed for IBA-1 immunofluorescence using 1.0 μm thick Z-sections in a confocal microscope revealed widespread accumulation of IN administered hiPSC-NSC-EVs into microglia in the forebrain, midbrain, and hindbrain. hiPSC-NSC-EVs accumulated inside the soma of ∼98% of microglia in both naïve and 5xFAD mice when examined at 45 min and 6 h post-IN administration. Examples of microglia that accumulated PKH26-labeled EVs (red particle aggregates) within soma at 45 min and 6 h post-IN administration in different brain regions are illustrated in Figures 6, 7. The illustrated brain regions include the OB (Figures 6A, G, M, S), mPFC (Figures 6B, H, N, T), SSC (Figures 6C, I, O, U), CA1 subfield of the hippocampus (Figures 6D, J, P, V), ECX (Figures 6 E, K, Q, W), and amygdala (Figures 6F, L, R, X), striatum (Figures 7A, G, M, S), TH (Figures 7B, H, N, T), HTH (Figures 7C, I, O, U), MB (Figures 7D, J, P, V), pons (Figures 7E, K, Q, W), and GCL-CBM (Figures 7F, L, R, X). Quantification revealed that at both 45 min and 6 h post-IN administration, the percentage of microglia incorporating PKH26 + hiPSC-NSC-EVs in different regions of the forebrain, midbrain and hindbrain were comparable between the naïve and 5xFAD groups (Figures 6, 7Y, Z). Thus, IN administration of 25 × 10^9^ hiPSC-NSC-EVs can target a vast majority of microglia in the entire brain of both naïve and 5xFAD mice within 45 min.

### 3.6. Analysis of orthogonal and 3-dimensional (3D) views of confocal Z-stacks confirmed that neurons and microglia in naïve and 5xFAD mice internalized PKH26 labeled hiPSC-NSC-EVs

**FIGURE 8:**
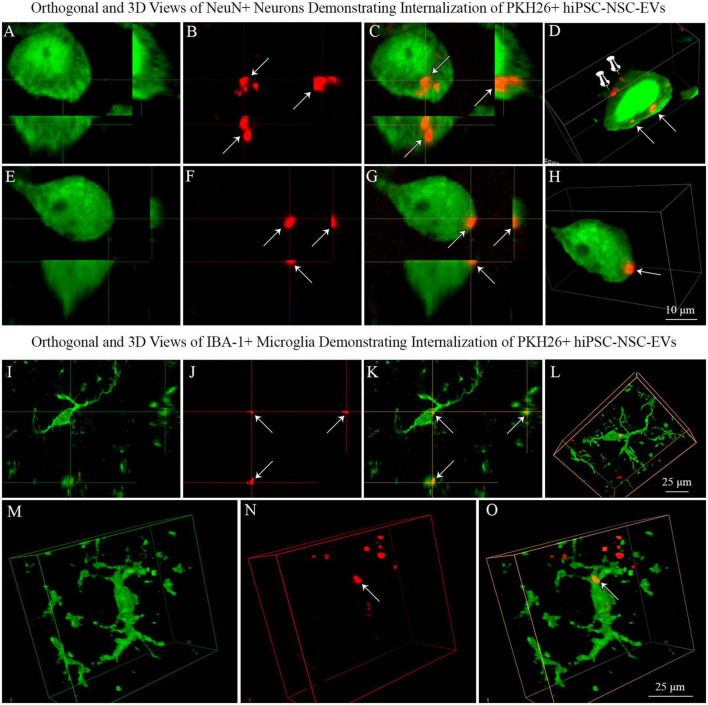
Neurons and microglia internalized PKH26 labeled hiPSC-NSC-EVs in 5xFAD mouse brain. Images **(A–C**,**E–G)** show orthogonal views of NeuN + neurons, demonstrating the internalization of PKH26 + hiPSC-NSC-EVs [arrows in panels **(A–C)**] or the partial entry of PKH26 + hiPSC-NSC-EVs into the cytoplasm of neurons [arrows in panel **(E–G)**]. Images **(D,H)** show 3-dimensional (3D) views of neurons shown in panels **(A–C,E–G)**, respectively. The neuron in panel **(D)** displays EVs incorporated into the cytoplasm (indicated by arrows) and EVs attached to the surface of the plasma membrane (indicated by thumb tacks). Scale bar, 10 μm. Images **(I–K)** show orthogonal views of IBA-1 + microglia exhibiting the internalization of PKH26 + hiPSC-NSC-EVs (arrows), whereas images **(L–O)** are 3-D views of microglia with PKH26 + hiPSC-NSC-EVs. Scale bar, 25 μm.

We generated orthogonal and 3D views of z-stacks of confocal images comprising neurons or microglia and IN-administered PKH26 + hiPSC-NSC-EVs. Such characterization confirmed that NeuN + neurons and IBA-1 + microglia internalized some PKH26 + hiPSC-NSC-EVs (Figures 8A–D, I–O). The analysis also demonstrated that some PKH26 + hiPSC-NSC-EVs were attached to the plasma membrane of neurons (Figure 8D) or in the process of entering into the cytoplasm of neurons (Figures 8E–H).

### 3.6. hiPSC-NSC-EV targeting varied based on both brain region and disease status at 45 min post-administration

**FIGURE 9:**
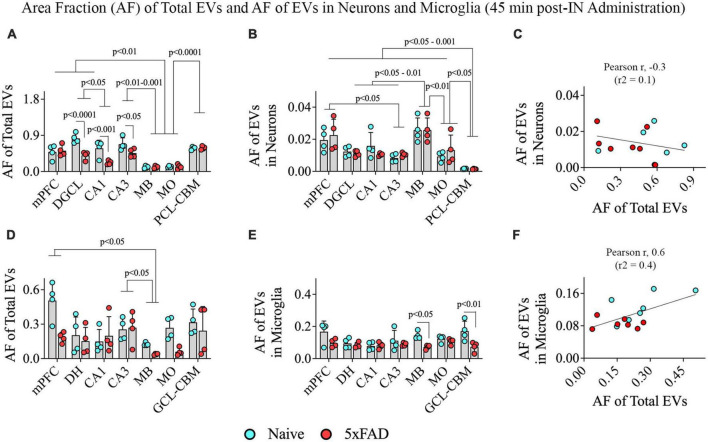
Distribution of hiPSC-NSC-EVs in neurons and microglia across different regions of the forebrain, midbrain, and hindbrain at 45 min post-intranasal administration in naïve and 5xFAD mice. The bar charts **(A,B,D,E)** compare the area fraction (AF) of total hiPSC-NSC-EVs and mean AF of hiPSC-NSC-EVs within neurons **(A,B)** and microglia **(D,E)** across different brain regions and between naïve and 5xFAD mice for each brain region. The bar charts **(C,F)** illustrate the correlation between AF of total EVs and AF of EVs in neurons **(C)** and between AF of total EVs and AF of EVs in microglia **(F)**. DGCL, dentate granule cell layer; DH, dentate hilus; GCL-CBM, granule cell layer of cerebellum; MB, midbrain; MO, medulla oblongata; mPFC, medial prefrontal cortex; PCL-CBM, Purkinje cell layer of cerebellum.

We performed two-way ANOVA analyses on the AF of all PKH26 + hiPSC-NSC-EVs within various grey matter regions of the brain. The extent of EV targeting varied based on the brain region and the disease status (*p* < 0.05–0.0001; Figure 9A). *Post hoc* analysis revealed that the AF of all EVs was higher in the mPFC, DGCL, and CA3 than the MB and MO (*p* < 0.01–0.001) and higher in the PCL-CBM than MB and MO (*p* < 0.0001). Overall, hiPSC-NSC-EV incorporation at 45 min post-IN administration was higher in forebrain regions than the MB and hindbrain regions, such as MO, in both naïve and 5xFAD mice. Furthermore, the AFs of all EVs were lower in the DGCL, CA1, and CA3 cell layers of 5xFAD mice compared to naïve mice (*p* < 0.05–0.0001, Figure 9A).

### 3.7. hiPSC-NSC-EV targeting into neurons and microglia varied only on the brain region at 45 min post-administration

Two-way ANOVA revealed differences in EV targeting into neurons based on the brain region (*p* < 0.001) but not on the disease status (*p* > 0.05; Figure 9B). Differences between different brain regions are highlighted in Figure 9B. One distinctive finding is that the extent of EV incorporation into neurons is higher in all regions of the forebrain, MB, and MO compared to the PCL-CBM (*p* < 0.05–0.001). However, the incorporation of hiPSC-NSC-EVs into neurons did not depend on the extent of targeted EVs in different brain regions, as there was no positive correlation between AF of total EVs and mean AF of EVs in neurons (Figure 9C). We also analyzed the AF of all PKH26 + hiPSC-NSC-EVs and the mean AF of EVs in microglia within various brain regions. The extent of EV targeting varied based on the brain region (*p* < 0.05) but not on the disease status (*p* > 0.05; Figure 9D). Differences between different brain regions are highlighted in Figure 9D. The mean AF of EVs within microglia (Figure 9E) was comparable across brain regions (*p* > 0.05) but was higher in naïve mice than in 5xFAD mice for some brain regions (*p* < 0.05–0.01). Furthermore, the incorporation of hiPSC-NSC-EVs into microglia did depend on the extent of targeted EVs in different brain regions, as there was a modest positive correlation (Pearson *r* = 0.6) between AF of total EVs and mean AF of EVs in microglia (Figure 9F). Thus, 5xFAD mice displayed reduced incorporation of EVs into cell layers in some forebrain regions.

### 3.8. hiPSC-NSC-EV targeting at 6 h post-administration varied based on both brain region and disease status

**FIGURE 10:**
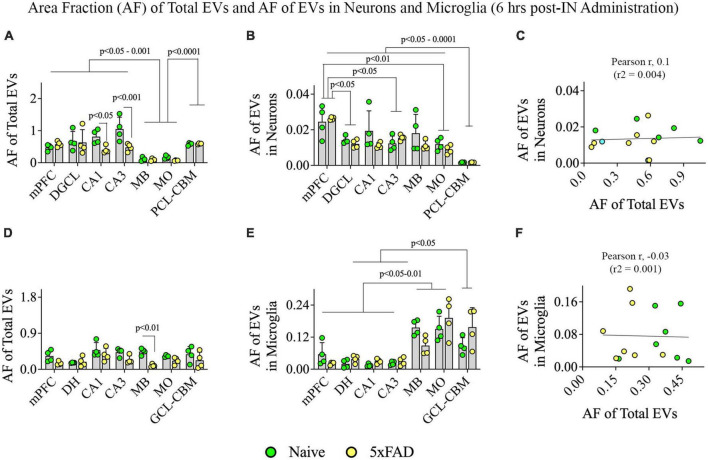
Distribution of hiPSC-NSC-EVs in neurons and microglia across different regions of the forebrain, midbrain, and hindbrain at 6 h post-intranasal administration in naïve and 5xFAD mice. The bar charts **(A,B,D,E)** compare the area fraction (AF) of total hiPSC-NSC-EVs and mean AF of hiPSC-NSC-EVs within neurons **(A,B)** and microglia **(D,E)** across different brain regions and between naïve and 5xFAD mice for each brain region. The bar charts **(C,F)** illustrate the correlation between AF of total EVs and AF of EVs in neurons **(C)** and between AF of total EVs and AF of EVs in microglia **(F)**. DGCL, dentate granule cell layer; DH, dentate hilus; GCL-CBM, granule cell layer of cerebellum; MB, midbrain; MO, medulla oblongata; mPFC, medial prefrontal cortex; PCL-CBM, Purkinje cell layer of cerebellum.

Two-way ANOVA revealed that the extent of EV targeting varied significantly based on the brain region and the disease status (*p* < 0.05–0.001; Figure 10A). *Post hoc* analysis revealed that the AF of all EVs was higher in the mPFC, DGCL, CA1, and CA3 cell layers than the MB and MO (*p* < 0.05–0.001) and higher in the PCL-CBM than MB and MO (*p* < 0.0001). Thus, hiPSC-NSC-EV incorporation at 6 h post-IN administration was higher in forebrain regions than the midbrain and hindbrain regions such as MO in both naïve and 5xFAD mice. The AFs of all EVs were lower in the CA1 and CA3 cell layers of 5xFAD mice compared to naïve mice (*p* < 0.05–0.001, Figure 10A).

### 3.9. hiPSC-NSC-EV targeting into neurons and microglia at 6 h post-administration varied only on the brain region

Two-way ANOVA revealed that the extent of EV targeting into neurons varied based on the brain region (*p* < 0.001) but not on disease status (*p* > 0.05; Figure 10B). Differences between different brain regions are highlighted in Figure 10B. However, the incorporation of hiPSC-NSC-EVs into neurons did not depend on the extent of targeted EVs in different brain regions, as there was no positive correlation between AF of total EVs and mean AF of EVs in neurons (Figure 10C).

Two-way ANOVA analyses revealed that the extent of total EV targeting did not vary based on the brain region (*p* > 0.05) but depended on the disease status, particularly in the MB (*p* < 0.01; Figure 10D). However, the mean AF of EVs within microglia (Figure 10E) varied based on the brain region (*p* < 0.0001) but not on the disease status (*p* > 0.05). Figure 10E highlights differences between different brain regions. Notably, hindbrain regions displayed higher incorporation of EVs into microglia than forebrain regions (Figure 10E). However, the incorporation of hiPSC-NSC-EVs into microglia did not depend on the extent of targeted EVs in different brain regions, as there was no positive correlation between AF of total EVs and mean AF of EVs in microglia (Figure 10F). Thus, 5xFAD mice displayed reduced incorporation of EVs into microglia in forebrain regions compared to midbrain and hindbrain regions.

### 3.10. hiPSC-NSC-EV incorporation into different brain regions, neurons, and microglia was mostly comparable between 45 min and 6 h post-administration in naïve and 5xFAD mice

**FIGURE 11:**
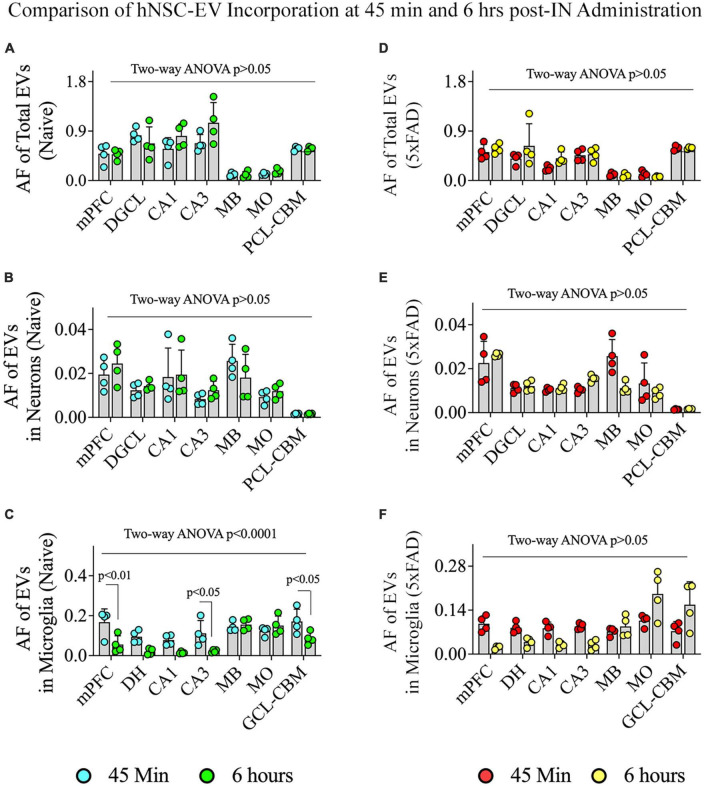
Comparison of the incorporation of hiPSC-NSC-EVs in neurons and microglia across different regions of the forebrain, midbrain, and hindbrain between 45 min and 6 h post-intranasal administration in naïve and 5xFAD mice. The bar charts **(A–C)** compare the area fraction (AF) of total hiPSC-NSC-EVs **(A)** and mean AF of hiPSC-NSC-EVs within neurons **(B)** and microglia **(C)** in naïve mice between 45 min and 6 h post-intranasal administration for each chosen brain region. The bar charts **(D–F)** compare the area fraction (AF) of total hiPSC-NSC-EVs **(D)** and mean AF of hiPSC-NSC-EVs within neurons **(E)** and microglia **(F)** in 5xFAD mice between 45 min and 6 h post-intranasal administration for each chosen brain region. DGCL, dentate granule cell layer; DH, dentate hilus; GCL-CBM, granule cell layer of cerebellum; MB, midbrain; MO, medulla oblongata; mPFC, medial prefrontal cortex; PCL-CBM, Purkinje cell layer of cerebellum.

We compared the incorporation of hiPSC-NSC-EVs into different brain regions, neurons, and microglia between 45 min and 6 h post-IN administration in naïve and 5xFAD mice (Figure 11). In naïve mice, the overall targeting of EVs into various brain regions and neurons in different brain regions were comparable between 45 min and 6 h post-IN administration (Figures 11A, B). However, the incorporation of EVs into microglia in different brain regions of naïve mice varied (*p* < 0.0001). Notably, mPFC, CA3 subfield, and GCL-CBM displayed reduced incorporation of EVs into microglia at 6 h compared to 45 min post-IN administration (*p* < 0.05–0.01, Figure 11C). In 5xFAD mice, the extent of EV incorporation into different brain regions, neurons, and microglia was comparable between 45 min and 6 h post-IN administration (*p* > 0.05, Figures 11D–F).

### 3.11. hiPSC-NSC-EVs incorporated into microglia surrounding amyloid-beta plaques in 5xFAD mice

**FIGURE 12:**
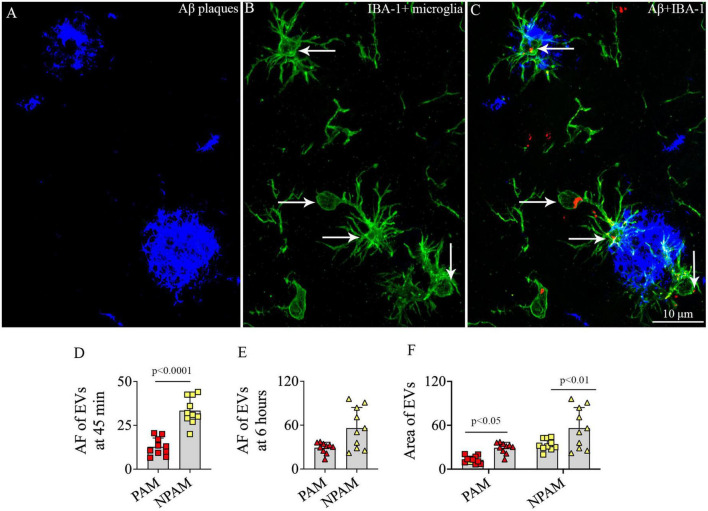
Intranasally administered hiPSC-NSC-EVs incorporate into plaque-associated microglia in 5xFAD mice **(A–C)**. The figure shows the incorporation of EVs into IBA-1 + plaque-associated microglia [arrows in panels **(B,C)**]. Scale bar, 10 μm. The bar charts **(D,E)** compare the mean area fraction of hNSC-EVs between plaque-associated microglia (PAM) and non-plaque associated microglia (NPAM) in the somatosensory cortex at 45 min and 6 h post-intranasal administration. Bar chart **(F)** compares the mean AF of EVs in PAM and NPAM between 45 min and 6 h post-intranasal administration.

To determine whether IN administered hiPSC-NSC-EVs could also get incorporated into PAM, we performed careful Z-section analysis of brain tissue sections processed for IBA-1 and Aβ dual immunofluorescence. Such analysis demonstrated that IN administered hiPSC-NSC-EVs do get incorporated into PAM surrounding Aβ plaques (Figures 12A–C). The accumulation of PKH26 + EVs within activated microglia resembled their accumulation within microglia found between plaques in the AD brain.

However, the comparison of AFs of EVs in PAM and NPAM at 45 min and 6 h post-IN administration revealed that the extent of incorporation of hiPSC-NSC-EVs is greater in NPAM vis-à-vis PAM, and the differences were significant at 45 min post-IN administration (*p* < 0.0001, Figures 12D–E). Furthermore, two-way ANOVA analysis of EV incorporation into PAM and NPAM at 45 min and 6 h post-IN administration revealed that EV incorporation into both PAM and NPAM increases between 45 min and 6 h post-IN administration (*p* < 0.05–0.01, Figure 12F).

### 3.12. IN administered hiPSC-NSC-EVs contacted processes of astrocytes in naive and 5xFAD mice

**FIGURE 13:**
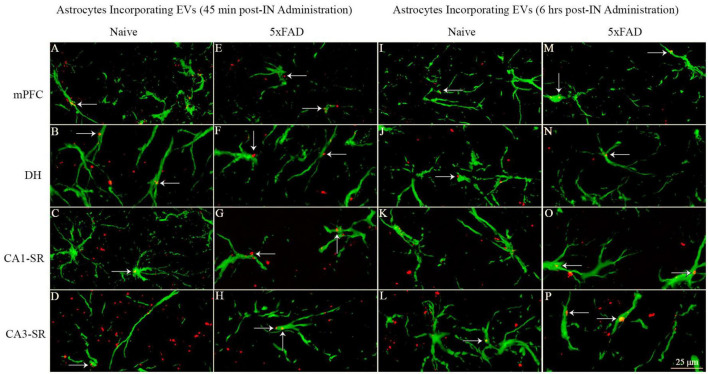
Intranasally administered hiPSC-NSC-EVs come in contact with GFAP + astrocyte processes and soma in naïve and 5xFAD mice. The figure illustrates the contact of EVs into GFAP + astrocyte processes and soma in different brain regions of naïve **(A–D, I–L)** and 5xFAD mice **(E–H, M–P)** at 45 min **(A–H)** and 6 h **(I–P)** post-administration. DH, dentate hilus; mPFC, medial prefrontal cortex; SR, stratum radiatum. Scale bar, 25 μm.

Probing of PKH26 + structures in serial brain tissue sections processed for GFAP immunofluorescence using 1.0 μm thick Z-sections in a confocal microscope revealed the apposition of IN administered hiPSC-NSC-EVs into astrocytic processes. hiPSC-NSC-EVs came in contact with the processes of the majority of astrocytes in both naïve and 5xFAD mice when examined at 45 min and 6 h post-IN administration. However, the incorporation of hiPSC-NSC-EVs into the soma of GFAP + astrocytes was rarely observed. Examples of astrocytic processes that made contact with the PKH26-labeled EVs (red particles) at 45 min and 6 h post-IN administration in different brain regions are illustrated in Figures 13A–P. The illustrated brain regions include mPFC, DH, and the stratum radiatum of CA1 and CA3 subfields. Thus, IN administration of 25 × 10^9^ hiPSC-NSC-EVs can target a vast majority of astrocytic processes in the brain of both naïve and 5xFAD mice within 45 min.

### 3.13. IN administered hiPSC-NSC-EVs contacted the soma of oligodendrocytes in white matter regions of naive and 5xFAD mice

**FIGURE 14:**
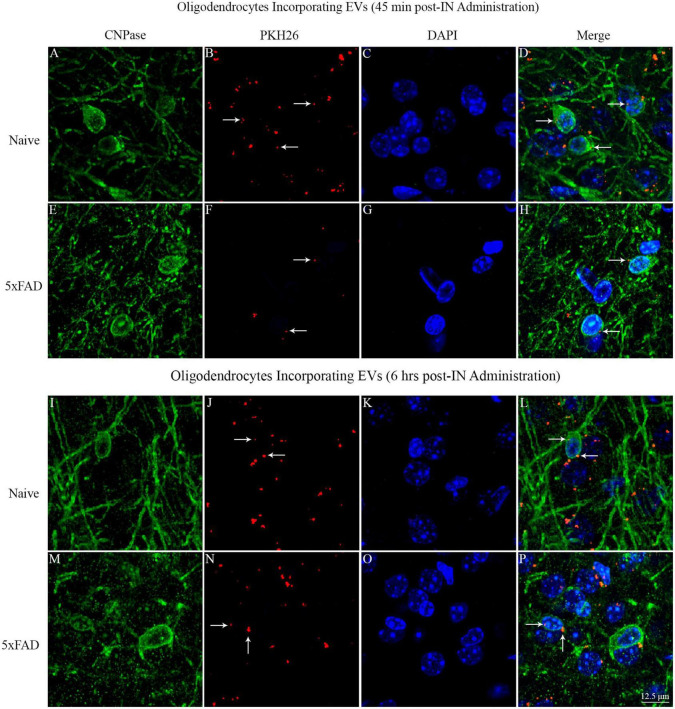
Intranasally administered hiPSC-NSC-EVs come in contact with the soma and processes of CNPase + oligodendrocytes in naïve and 5xFAD mice. The figure illustrates the contact of EVs into CNPase + soma and processes of oligodendrocytes in different brain regions of naïve **(A–D,I–L)** and 5xFAD mice **(E–H,M–P)** at 45 min **(A–H)** and 6 h **(I–P)** post-administration. Scale bar, 12.5 μm.

Investigation of PKH26 + structures in serial brain tissue sections processed for CNPase immunofluorescence using 0.5 μm thick Z-sections in a confocal microscope revealed the apposition of IN administered hiPSC-NSC-EVs into the soma of oligodendrocytes in the white matter regions of the brain. hiPSC-NSC-EVs came in contact with the soma of most oligodendrocytes in both naïve and 5xFAD mice when examined at 45 min and 6 h post-IN administration. However, the incorporation of hiPSC-NSC-EVs inside the soma of CNPase + oligodendrocytes was rarely observed. Examples of the soma of CNPase + oligodendrocytes that made contact with the PKH26-labeled EVs (red particles) at 45 min and 6 h post-IN administration in the corpus callosum are illustrated in Figure 14. Thus, IN administration of hiPSC-NSC-EVs can target the soma of oligodendrocytes in white matter regions of the brain in both naïve (Figures 14A–D, I–L) and 5xFAD mice (Figures 14E–H, M–P) within 45 min after IN administration.

## 4. Discussion

This study provides novel evidence that IN administration is an efficient approach for delivering therapeutic hiPSC-NSC-EVs into neurons, microglia, astrocytes, and oligodendrocytes in virtually all regions of the forebrain, midbrain, and hindbrain of both naïve and 5xFAD mice. In the case of neurons and microglia, PKH26-labeled EVs were incorporated into their cytoplasm. In contrast, in astrocytes and oligodendrocytes, EVs came in contact with the plasma membrane of their soma or processes but were only occasionally seen inside the soma. Furthermore, measurement of neurons and microglia incorporating EVs in different brain regions demonstrated that ∼98% of neurons and microglia internalized EVs in both naïve and 5xFAD mice. AF analysis revealed that, in both naïve and 5xFAD mice, higher fractions of hiPSC-NSC-EVs incorporate into forebrain regions than the midbrain and hindbrain regions at both 45 min and 6 h post-IN administration, implying that IN-administered EVs can access rostral brain regions more rapidly than caudal brain regions. Moreover, compared to naïve mice, 5xFAD mice displayed a reduced fraction of hiPSC-NSC-EVs within cell layers in forebrain regions and within microglia in midbrain and some hindbrain regions at 45 min post-IN administration, suggesting that disease status likely reduces the extent of incorporation of EVs into distinct brain regions and microglia. Also, in 5xFAD mice, the extent of EV incorporation into different brain regions, neurons, and microglia was comparable between 45 min and 6 h post-IN administration, implying that most IN-administered EVs reach their brain targets by 45 min.

### 4.1. Technical considerations linked to PKH26 labeling of hiPSC-NSC-EVs

This study employed PKH26 to label hiPSC-NSC-EVs to compare the biodistribution of EVs between naïve and 5xFAD mice in the early stage of the amyloidosis. Since PKH26 tends to aggregate, the possible presence of free PKH26 aggregates in labeled EV preparations was evaluated by performing a cell culture study. For this, neural stem cell cultures were incubated for an hour with PKH26-labeled EVs or PKH26 dye solution, undergoing the identical incubation and filtration steps as the PKH26-labeled EVs. The findings showed PKH26 + structures (red particles) in neural stem cell cultures incubated with PKH26-labeled EVs but not in neural stem cell cultures incubated with PKH26 dye solution undergoing the identical incubation and filtration steps as the PKH26-labeled EVs, confirming that the procedure employed in the study resulted in a suspension of PKH26-labeled EVs containing minimal or no free PKH26 dye. Furthermore, this study determined whether neurons, microglia, astrocytes, and oligodendrocytes in various regions of the forebrain, midbrain, and hindbrain take up IN-administered PKH26-labeled hiPSC-NSC-EVs at 45 min and 6 h post-administration. Most NeuN + neurons and IBA-1 + microglia displayed PKH26 + structures (red dots or aggregates) within their soma at both time points. Furthermore, PKH26 + structures were consistently seen in contact with the plasma membranes of the soma and processes of astrocytes and oligodendrocytes at both time points. The brain tissue sections were investigated with Z-sectioning in a confocal microscope following dual immunofluorescence staining for a neural cell marker with CD63 or CD81 to validate whether red particles found within neural cells are IN-administered hiPSC-NSC-EVs or dye particles. Such analysis revealed that all PKH26 + structures within neural cells expressed CD63 and CD81, confirming that IN-administered EVs target neural cells throughout the brain.

### 4.2. Possible routes by which IN administered EVs quickly permeated the brain

This study demonstrated that IN-administered PKH26-labeled hiPSC-NSC-EVs quickly enter the entire brain in naïve and 5xFAD mice. Targeting of EVs into neurons and microglia, astrocytes, and oligodendrocytes was mostly comparable at 45 min and 6 h post-administration in both naïve and 5xFAD mice. These results have significance because earlier studies have suggested that intravenously (IV) administered EVs accumulate predominantly in the liver and spleen, resulting in a small fraction of IV-administered EVs reaching the brain (Lai et al., 2014; Wiklander et al., 2015; Kang et al., 2021; Laìzaro-Ibaìnþez et al., 2021; Pauwels et al., 2021). A previous study comparing the biodistribution of stem cell-derived EVs in the brain following IN or IV administration has also reported superior brain accumulation of EVs with IN administration (Betzer et al., 2017). The efficacy of IN administration of EVs to disseminate into different neural cell types in the entire brain is highly beneficial because it provides a route for simple, painless, non-invasive, repeated delivery of EVs into the brain. Besides increasing the bioavailability of EVs in the brain, IN administration also reduces the accumulation and potential adverse effects in organs such as the liver and spleen.

How do IN-administered EVs enter different regions of the forebrain, midbrain, and hindbrain so quickly? One route by which IN-administered EVs could enter the brain is through neurons of the olfactory bulb. Such entry would involve EV internalization by olfactory sensory neurons in the olfactory epithelium through endocytosis and then transsynaptic transportation of EVs *via* olfactory nerves into olfactory bulb neurons and then into other brain regions. Such transport is feasible, as EVs can travel *via* axons by hijacking the endosomal system (Lochhead and Thorne, 2012; Polanco et al., 2018). However, while a certain fraction of EVs can enter certain brain regions through such transsynaptic routes, it is improbable that IN-administered EVs could permeate the entire brain within 45 min through this route alone. In this study, at both 45 min and 6 h post-IN administration, the percentage of neurons and microglia incorporating EVs within the olfactory bulb (the most rostral region of the brain) did not differ from the percentage of neurons and microglia incorporating EVs within the entorhinal cortex, medulla oblongata or the cerebellum (the caudal regions of the brain), implying that IN administrated EVs concurrently invaded the entire brain region. In this context, the possibility of EVs taking the extracellular route, involving the perineural movement of EVs along olfactory nerves passing through the cribriform plate into the subarachnoid space, is highly likely (Lochhead and Davis, 2019). Through this extracellular route, a larger fraction of IN-administered EVs can quickly access the subarachnoid space. Several studies have demonstrated a communication between the nasal lymph compartment in the submucosa of olfactory and respiratory epithelium and the cerebrospinal fluid (CSF) compartment of the brain (Johnston et al., 2004; Zakharov et al., 2004). It is possible that because of the permeabilization of the nasal mucous membrane through hyaluronidase, EVs first enter the nasal lymph compartment and then reach the CSF-filled subarachnoid space covering the surface of the entire brain.

Once EVs permeate the subarachnoid space, CSF flow can facilitate their spread over the brain’s entire surface and into brain ventricles, allowing EVs to enter deeper brain regions *via* the interstitial space (ISS). Previous studies have shown that subarachnoid CSF rapidly passes into deeper brain regions alongside the perivascular spaces and reaches the level of capillaries (Iliff et al., 2012). Then, aquaporin-4 channels positioned on the perivascular end-feet of astrocytes enable the convective flow of CSF into the ISS, resulting in the mixing of CSF with the interstitial fluid (ISF) (Iliff et al., 2012). Since the ability of EVs to travel *via* body fluids such as CSF is well known (Pauwels et al., 2021), IN-administered EVs likely permeate the entire ISS quickly through the above routes. From the ISF, neurons can take up EVs *via* endocytosis, and microglia can take up EVs through macropinocytosis or phagocytosis. In contrast, the remaining EVs contact the plasma membrane of the soma and processes of astrocytes and oligodendrocytes. Additionally, EVs might enter the systemic circulation from the nasal epithelium as the nasal mucous membrane is highly vascular (Harkema et al., 2012), and EV penetrance into endothelial cells is typically high (Herman et al., 2021). From the systemic circulation, EVs can cross the blood-brain and blood-CSF barriers and enter the brain (Herman et al., 2021). Thus, IN administration provides multiple options for EVs to quickly target neurons, microglia, astrocytes, and oligodendrocytes in all brain regions. In this context, the finding from our AF analysis is of interest. In both naïve and 5xFAD mice, higher fractions of hiPSC-NSC-EVs incorporate into forebrain regions than the midbrain and hindbrain regions at 45 min and 6 h post-IN administration. Such a finding suggests that IN-administered EVs enter forebrain regions through more than one route, which may include their transport *via* olfactory nerves and through CSF flow into the ISS.

### 4.3. Neurons and microglia internalized large amounts of hiPSC-NSC-EVs in both naïve and 5xFAD mice

EVs can enter cells through several mechanisms, including direct fusion with the plasma membrane, clathrin-dependent, caveolin-1 (Cav-1)-dependent, lipid raft-dependent endocytosis, micropinocytosis, and phagocytosis (Théry et al., 2009; Colombo et al., 2014; Mulcahy et al., 2014). In this study, a significant amount of IN-administered EVs were incorporated into the cytoplasm of neurons in virtually all regions of the forebrain, midbrain, and hindbrain in both naïve and 5xFAD mice. Furthermore, excitatory neurons (e.g., neurons in the hippocampal dentate granule cell layer, CA1 and CA3 pyramidal neurons, and large pyramidal neurons in the cerebral cortex) and inhibitory interneurons (expressing parvalbumin or neuropeptide Y) exhibited a similar ability to internalize hiPSC-NSC-EVs. How do neurons internalize EVs? A study has shown that Cav-1 serves as a receptor for the endocytosis of EVs by neurons (Yue et al., 2019). Cav-1, a membrane/lipid raft scaffolding protein required for synaptic and neural plasticity (Wang et al., 2021a), undergoes upregulation following insults such as oxygen-glucose deprivation, resulting in increased uptake of EVs by neurons (Yue et al., 2019). Moreover, the knockdown of Cav-1 reduces EV intake by neurons, consistent with the observations that interference of endocytosis-related molecules would reduce the internalization of EVs by cells (Costa Verdera et al., 2017). In this study, the incorporation of IN-administered EVs by neurons in multiple brain regions was mostly similar between naïve mice and 3 months old 5xFAD mice. Such observation implies that EV intake ability *via* endocytosis did not significantly alter in 5xFAD mice at 3 months. While age-related Cav-1 expression alterations in 5xFAD mice are unknown, a study has shown that Cav-1 expression decreases at 9 months of age in the PSAPP-Tg (*APPSwePS1d9)* mouse model of AD (Wang et al., 2021b). Therefore, it remains to be investigated whether a similar extent of EV incorporation would occur in neurons in 5xFAD mice at a relatively advanced age associated with increased disease pathogenesis and cognitive and mood impairments.

In this study, a large amount of IN-administered EVs aggregated within the cytoplasm of microglia in virtually all regions of the forebrain, midbrain, and hindbrain in both naïve and 5xFAD mice. A previous study has suggested that, both *in vitro* and *in vivo*, the uptake of EVs by microglia involves macropinocytosis. Macropinocytosis is a process that involves the actin-driven extension of plasma membrane ruffles forming a cup-like structure around EVs, followed by the fusion of their distal tips leading to a macropinosome containing EVs within microglia (Fitzner et al., 2011; Canton, 2018). Then, the generation of reactive oxygen species likely damages the integrity of the macropinosome membrane through lipid peroxidation leading to the release of EVs into the cytosol (Canton, 2018). Microglia can also take up EVs *via* phagocytosis (Ogaki et al., 2021). The incorporation of a large number of hiPSC-NSC-EVs by microglia in the AD brain is likely beneficial because of the robust antiinflammatory properties of hiPSC-NSC-EVs. NSC-EVs have robust immunomodulatory activity in target cells, which makes them attractive for application in conditions such as neurodegenerative diseases (Cossetti et al., 2014; Willis et al., 2020b). Furthermore, a previous study has shown that hiPSC-NSC-EVs can attenuate the proinflammatory cytokine interleukin-6 (IL-6) production by macrophages stimulated with lipopolysaccharide (Upadhya et al., 2020a). Moreover, hiPSC-NSCs reduced the release of proinflammatory cytokines tumor necrosis factor-alpha (TNF-α) and interleukin-1 beta (IL-1b) by human microglial cells stimulated with lipopolysaccharide (Upadhya et al., 2022). This study also demonstrated that hiPSC-NSC-EVs get incorporated into PAM. However, the comparison of AFs of EVs in PAM and NPAM at 45 min and 6 h post-IN administration demonstrated that the overall extent of incorporation of hiPSC-NSC-EVs is greater in NPAM vis-à-vis PAM, which might reflect the somewhat compromised ability of PAM for macropinocytosis and phagocytosis. Nonetheless, the ability of hiPSC-NSC-EVs to enter PAM is likely beneficial for transforming PAM into less inflammatory microglia. However, it remains to be investigated whether microglia incorporating hiPSC-NSC-EVs would undergo significant transcriptomic changes leading to their transformation into a less proinflammatory state in 5xFAD mice.

### 4.4. Astrocytes and oligodendrocytes rarely internalized hiPSC-NSC-EVs

IN administered PKH26-labeled hiPSC-NSC-EVs came in contact with the plasma membrane of processes and soma of astrocytes and oligodendrocytes throughout the brain of naïve and 5xFAD mice. However, internalization of EVs by these neural cells was rarely seen, implying that the mode of transfer of EV cargo into astrocytes and oligodendrocytes differs from the transfer of EV cargo into neurons and microglia in the brain. While many *in vitro* studies have demonstrated the uptake of a variety of EVs by cultured astrocytes, demonstration of EV uptake by astrocytes *in vivo* is rare (Ogaki et al., 2021). It has been suggested that only occasional uptake of exogenously administered EVs by astrocytes *in vivo* reflects their tendency in the brain to secrete more EVs rather than uptake EVs (Verkhratsky et al., 2016; Ogaki et al., 2021). Furthermore, no studies have demonstrated EV internalization by oligodendrocytes in the brain. However, oligodendrocyte progenitor cells can internalize EVs *in vitro* (Kurachi et al., 2016; Osawa et al., 2017). Also, EVs secreted by circulating immune cells have been shown to increase myelin content in hippocampal slice cultures (Pusic et al., 2016). Thus, astrocytes and oligodendrocytes rarely internalized hiPSC-NSC-EVs in naïve and 5xFAD mice. Nonetheless, one cannot rule out the possibility of transferring EV cargo into the cytosol of astrocytes and oligodendrocytes through the fusion of plasma membranes. Such a scenario is likely because stem cell-derived EVs have improved the function of astrocytes *in vivo*. For example, miR-138-5p-overexpressing stem cell-derived EVs prevented apoptosis of astrocytes following ischemic stroke by targeting lipocalin 2 (Deng et al., 2019), and miR-146a overexpressing stem cell-derived EVs reduced inflammation by damaged astrocytes in a rat model of diabetes (Kubota et al., 2018) and transfer of miR-133b from stem cell-derived EVs into astrocytes reduced glial scar formation in a stroke model (Xin et al., 2013). In this study, EV contact by the plasma membrane of processes and soma of astrocytes and oligodendrocytes in multiple brain regions did not differ between naïve mice and 3 months old 5xFAD mice, implying that interactions with EVs by astrocytes and oligodendrocytes did not alter in AD mice at 3 months of age.

## 5. Conclusion

This study demonstrated that IN administration of hiPSC-NSC-EVs leads to their incorporation into the cytosol of neurons and microglia and plasma membranes of the soma and processes of astrocytes and oligodendrocytes in all regions of the forebrain, midbrain, and hindbrain in both naïve and 5xFAD mice. The ability to target therapeutic hiPSC-NSC-EVs into different cell types in virtually all brain regions has considerable significance for treating AD, as the disease involves neuroinflammation, synapse loss, and neurodegeneration in multiple regions. Importantly, IN-administered hiPSC-NSC-EVs targeted the different neural cell types in naïve and 5xFAD mice. Such behavior of hiPSC-NSC-EVs in 5xFAD mice demonstrated their ability to target different neural cell types in the brain during an early stage of amyloidosis as 3 months old 5xFAD mice were employed in this study, which is a stage of AD displaying only modest deposition of amyloid-beta and neuroinflammation with no cognitive impairments. Therefore, future studies need to investigate the pattern of EV biodistribution occurring in different brain regions and cell types in 5xFAD mice at advanced stages of the disease. Furthermore, since the current biodistribution study employed xenogeneic EVs, further studies will be needed to determine whether allogeneic and xenogeneic EVs behave similarly following IN administration.

## Data availability statement

The raw data supporting the conclusions of this article will be made available by the authors, without undue reservation.

## Ethics statement

The animal study was reviewed and approved by the Animal Care and Use Committee at Texas A&M University.

## Author contributions

AS: concept. AS, SA, RU, MKo, LM, and SR: research design. SA, MKo, SR, and AS: preparation of figure composites. SA and AS: manuscript writing. All authors contributed to the data collection, analysis, and interpretation and provided the feedback, edits, and additions to the manuscript text and approved the final version of the manuscript.

## References

[B1] Abels E. R. Breakefield X. O. (2016). Introduction to extracellular vesicles: Biogenesis, RNA cargo selection, content, release, and uptake. *Cell Mol. Neurobiol.* 36 301–312. 10.1007/s10571-016-0366-z 27053351 PMC5546313

[B2] Ager R. R. Davis J. L. Agazaryan A. Benavente F. Poon W. W. LaFerla F. M. (2015). Human neural stem cells improve cognition and promote synaptic growth in two complementary transgenic models of Alzheimer’s disease and neuronal loss. *Hippocampus* 25 813–826. 10.1002/hipo.22405 25530343 PMC4722865

[B3] Bambakidis T. Dekker S. E. Williams A. M. Biesterveld B. E. Bhatti U. F. Liu B. (2022). Early treatment with a single dose of mesenchymal stem cell derived extracellular vesicles modulates the brain transcriptome to create neuroprotective changes in a porcine model of traumatic brain injury and hemorrhagic shock. *Shock* 57 281–290. 10.1097/shk.0000000000001889 34798633

[B4] Bedel A. Beliveau F. Lamrissi-Garcia I. Rousseau B. Moranvillier I. Rucheton B. (2017). Preventing pluripotent cell teratoma in regenerative medicine applied to hematology disorders. *Stem Cells Transl. Med*. 6 382–393. 10.5966/sctm.2016-0201 28191782 PMC5442801

[B5] Betzer O. Shilo M. Opochinsky R. Barnoy E. Motiei M. Okun E. (2017). The effect of nanoparticle size on the ability to cross the blood–brain barrier: An in vivo study. *Nanomedicine* 12 1533–1546. 10.2217/nnm-2017-0022 28621578

[B6] Blurton-Jones M. Kitazawa M. Martinez-Coria H. Castello N. A. Müller F. J. Loring J. F. (2009). Neural stem cells improve cognition via BDNF in a transgenic model of Alzheimer disease. *Proc. Natl. Acad. Sci. U. S. A.* 106 13594–13599. 10.1073/pnas.0901402106 19633196 PMC2715325

[B7] Cai G. Cai G. Zhou H. Zhuang Z. Liu K. Pei S. (2021). Mesenchymal stem cell-derived exosome miR-542-3p suppresses inflammation and prevents cerebral infarction. *Stem Cell Res. Ther*. 12:2. 10.1186/s13287-020-02030-w 33407827 PMC7786953

[B8] Campos H. C. Ribeiro D. E. Hashiguchi D. Hukuda D. Y. Gimenes C. Romariz S. A. A. (2022). Distinct effects of the hippocampal transplantation of neural and mesenchymal stem cells in a transgenic model of Alzheimer’s disease. *Stem Cell Rev. Rep*. 18 781–791. 10.1007/s12015-021-10321-9 34997526

[B9] Canton J. (2018). Macropinocytosis: New insights into its underappreciated role in innate immune cell surveillance. *Front. Immunol*. 2:2286. 10.3389/fimmu.2018.02286 30333835 PMC6176211

[B10] Chen H. X. Liang F. C. Gu P. Xu B. L. Xu H. J. Wang W. T. (2020). Exosomes derived from mesenchymal stem cells repair a Parkinson’s disease model by inducing autophagy. *Cell Death Dis*. 11:288. 10.1038/s41419-020-2473-5 32341347 PMC7184757

[B11] Colombo M. Raposo G. Théry C. (2014). Biogenesis, secretion, and intercellular interactions of exosomes and other extracellular vesicles. *Annu. Rev. Cell. Dev. Biol*. 30 255–289. 10.1146/annurev-cellbio-101512-122326 25288114

[B12] Cone A. S. Yuan X. Sun L. Duke L. C. Vreones M. P. Carrier A. N. (2021). Mesenchymal stem cell-derived extracellular vesicles ameliorate Alzheimer’s disease-like phenotypes in a preclinical mouse model. *Theranostics* 11 8129–8142. 10.7150/thno.62069 34373732 PMC8344012

[B13] Cossetti C. Iraci N. Mercer T. R. Leonardi T. Alpi E. Drago D. (2014). Extracellular vesicles from neural stem cells transfer IFN-γ via Ifngr1 to activate stat1 signaling in target cells. *Mol. Cell* 56 193–204. 10.1016/j.molcel.2014.08.020 25242146 PMC4578249

[B14] Costa Verdera H. Gitz-Francois J. J. Schiffelers R. M. Vader P. (2017). Cellular uptake of extracellular vesicles is mediated by clathrin-independent endocytosis and macropinocytosis. *J. Control. Release* 266 100–108. 10.1016/j.jconrel.2017.09.019 28919558

[B15] Deng Y. Chen D. Gao F. Lv H. Zhang G. Sun X. (2019). Exosomes derived from microRNA-138-5p-overexpressing bone marrow-derived mesenchymal stem cells confer neuroprotection to astrocytes following ischemic stroke via inhibition of LCN2. *J. Biol. Eng.* 13:71. 10.1186/s13036-019-0193-0 31485266 PMC6714399

[B16] Drago D. Cossetti C. Iraci N. Gaude E. Musco G. Bachi A. (2013). The stem cell secretome and its role in brain repair. *Biochimie* 95 2271–2285. 10.1016/j.biochi.2013.06.020 23827856 PMC4061727

[B17] Fitzgerald M. Sotuyo N. Tischfield D. J. Anderson S. A. (2020). Generation of cerebral cortical GABAergic interneurons from pluripotent stem cells. *Stem Cells* 38 1375–1386. 10.1002/stem.3252 32638460

[B18] Fitzner D. Schnaars M. van Rossum D. Krishnamoorthy G. Dibaj P. Bakhti M. (2011). Selective transfer of exosomes from oligodendrocytes to microglia by macropinocytosis. *J. Cell Sci.* 124 447–458. 10.1242/jcs.074088 21242314

[B19] Fricker R. A. Carpenter M. K. Winkler C. Greco C. Gates M. A. Björklund A. (1999). Site-specific migration and neuronal differentiation of human neural progenitor cells after transplantation in the adult rat brain. *J. Neurosci*. 19 5990–6005. 10.1523/jneurosci.19-14-05990.1999 10407037 PMC6783093

[B20] Frozza R. L. Lourenco M. V. De Felice F. G. (2018). Challenges for Alzheimer’s disease therapy: Insights from novel mechanisms beyond memory defects. *Front. Neurosci*. 12:37. 10.3389/fnins.2018.00037 29467605 PMC5808215

[B21] Fujiwara N. Shimizu J. Takai K. Arimitsu N. Saito A. Kono T. (2013). Restoration of spatial memory dysfunction of human APP transgenic mice by transplantation of neuronal precursors derived from human iPS cells. *Neurosci. Lett.* 557 129–134. 24466594

[B22] Han F. Bi J. Qiao L. Arancio O. (2020). Stem cell therapy for Alzheimer’s disease. *Adv. Exp. Med. Biol*. 1266 39–55. 10.1007/978-981-15-4370-8_4 33105494

[B23] Harkema J. R. Carey S. A. Wagner J. G. Dintzis S. M. Liggitt D. (2012). “Nose, Sinus, Pharynx, and Larynx,” in *Comparative Anatomy and Histology*, eds Treuting P. M. Dintzis S. M. Montine K. S. (San Diego: Academic Press), 71–94.

[B24] Herman S. Fishel I. Offen D. (2021). Intranasal delivery of mesenchymal stem cells-derived extracellular vesicles for the treatment of neurological diseases. *Stem Cells* 39 1589–1600. 10.1002/stem.3456 34520591

[B25] Hill A. F. (2019). Extracellular vesicles and neurodegenerative diseases. *J. Neurosci.* 39 9269–9273. 10.1523/jneurosci.0147-18.2019 31748282 PMC6867808

[B26] Hu Y. Qu Z. Y. Cao S. Y. Li Q. Ma L. Krencik R. (2016). Directed differentiation of basal forebrain cholinergic neurons from human pluripotent stem cells. *J. Neurosci. Methods* 266 42–49. 10.1016/j.jneumeth.2016.03.017 27036311

[B27] Iliff J. J. Wang M. Liao Y. Plogg B. A. Peng W. Gundersen G. A. (2012). A paravascular pathway facilitates csf flow through the brain parenchyma and the clearance of interstitial solutes, including amyloid β. *Sci. Transl. Med*. 4:147ra111. 10.1126/scitranslmed.3003748 22896675 PMC3551275

[B28] Itakura G. Kawabata S. Ando M. Nishiyama Y. Sugai K. Ozaki M. (2017). Fail-safe system against potential tumorigenicity after transplantation of ipsc derivatives. *Stem Cell Rep.* 8 673–684. 10.1016/j.stemcr.2017.02.003 28262544 PMC5355810

[B29] Johnston M. Zakharov A. Papaiconomou C. Salmasi G. Armstrong D. (2004). Evidence of connections between cerebrospinal fluid and nasal lymphatic vessels in humans, non-human primates and other mammalian species. *Cereb. Fluid Res.* 1:2. 10.1186/1743-8454-1-2 15679948 PMC546409

[B30] Kang M. Jordan V. Blenkiron C. Chamley L. W. (2021). Biodistribution of extracellular vesicles following administration into animals: A systematic review. *J. Extracell. Vesicles* 10:e12085. 10.1002/jev2.12085 34194679 PMC8224174

[B31] Katabathina V. Menias C. O. Pickhardt P. Lubner M. Prasad S. R. (2016). Complications of immunosuppressive therapy in solid organ transplantation. *Radiol. Clin. North Am*. 54 303–319. 10.1016/j.rcl.2015.09.009 26896226

[B32] Kim D. K. Nishida H. An S. Y. Shetty A. K. Bartosh T. J. Prockop D. J. (2016). Chromatographically isolated CD63+CD81+ extracellular vesicles from mesenchymal stromal cells rescue cognitive impairments after TBI. *Proc. Natl. Acad. Sci. U. S. A.* 113 170–175. 10.1073/pnas.1522297113 26699510 PMC4711859

[B33] Kiskinis E. Eggan K. (2010). Progress toward the clinical application of patient-specific pluripotent stem cells. *J. Clin. Invest*. 120 51–59. 10.1172/jci40553 20051636 PMC2798698

[B34] Kodali M. Castro O. W. Kim D. K. Thomas A. Shuai B. Attaluri S. (2019). Intranasally administered human MSC-derived extracellular vesicles pervasively incorporate into neurons and microglia in both intact and status epilepticus injured forebrain. *Int. J. Mol. Sci*. 21:181. 10.3390/ijms21010181 PMC6981466 31888012

[B35] Kodali M. Castro O. W. Kim D. K. Thomas A. Shuai B. Attaluri S. (2020). Intranasally administered human MSC-derived extracellular vesicles pervasively incorporate into neurons and microglia in both intact and status epilepticus injured forebrain. *Int. J. Mol. Sci*. 21:181. 10.3390/ijms21010181 PMC6981466 31888012

[B36] Kodali M. Madhu L. N. Reger R. L. Milutinovic B. Upadhya R. Gonzalez J. J. (2023). Intranasally administered human MSC-derived extracellular vesicles inhibit NLRP3-p38/MAPK signaling after TBI and prevent chronic brain dysfunction. *Brain Behav. Immun*. 108 118–134. 10.1016/j.bbi.2022.11.014 36427808 PMC9974012

[B37] Kuang Y. Zheng X. Zhang L. Ai X. Venkataramani V. Kilic E. (2020). Adipose-derived mesenchymal stem cells reduce autophagy in stroke mice by extracellular vesicle transfer of miR-25. *J. Extracell. Vesicles* 10:e12024. 10.1002/jev2.12024 33304476 PMC7710129

[B38] Kubota K. Nakano M. Kobayashi E. Mizue Y. Chikenji T. Otani M. (2018). An enriched environment prevents diabetes-induced cognitive impairment in rats by enhancing exosomal miR-146a secretion from endogenous bone marrow-derived mesenchymal stem cells. *PLoS One* 13:e0204252. 10.1371/journal.pone.0204252 30240403 PMC6150479

[B39] Kurachi M. Mikuni M. Ishizaki Y. (2016). Extracellular vesicles from vascular endothelial cells promote survival, proliferation and motility of oligodendrocyte precursor cells. *PLoS One* 11:e0159158. 10.1371/journal.pone.0159158 27403742 PMC4942096

[B40] Lai C. P. Mardini O. Ericsson M. Prabhakar S. Maguire C. A. Chen J. W. (2014). Dynamic biodistribution of extracellular vesicles in vivo using a multimodal imaging reporter. *ACS Nano* 8 483–494. 10.1021/nn404945r 24383518 PMC3934350

[B41] Laìzaro-Ibaìnþez E. Faruqu F. N. Saleh A. F. Silva A. M. Tzu-Wen W. Rak J. (2021). Selection of fluorescent, bioluminescent, and radioactive tracers to accurately reflect extracellular vesicle biodistribution in vivo. *ACS Nano* 15 3212–3227. 10.1021/acsnano.0c09873 33470092 PMC7905875

[B42] Lee E. J. Choi Y. Lee H. J. Hwang D. W. Lee D. S. (2022). Human neural stem cell-derived extracellular vesicles protect against Parkinson’s disease pathologies. *J. Nanobiotechnol.* 20:198. 10.1186/s12951-022-01356-2 35468855 PMC9040239

[B43] Levi A. D. Anderson K. D. Okonkwo D. O. Park P. Bryce T. N. Kurpad S. N. (2019). Clinical outcomes from a multi-center study of human neural stem cell transplantation in chronic cervical spinal cord injury. *J. Neurotrauma* 36 891–902. 10.1089/neu.2018.5843 30180779

[B44] Li X. Zhu H. Sun X. Zuo F. Lei J. Wang Z. (2016). Human neural stem cell transplantation rescues cognitive defects in APP/PS1 model of Alzheimer’s disease by enhancing neuronal connectivity and metabolic activity. *Front. Aging Neurosci*. 8:282. 10.3389/fnagi.2016.00282 27932977 PMC5120101

[B45] Liu Q. Tan Y. Qu T. Zhang J. Duan X. Xu H. (2020). Therapeutic mechanism of human neural stem cell-derived extracellular vesicles against hypoxia-reperfusion injury in vitro. *Life Sci*. 254:117772. 10.1016/j.lfs.2020.117772 32437794

[B46] Liu Y. Liu H. Sauvey C. Yao L. Zarnowska E. D. Zhang S. C. (2013). Directed differentiation of forebrain GABA interneurons from human pluripotent stem cells. *Nat. Protoc*. 8 1670–1679. 10.1038/nprot.2013.106 23928500 PMC4121169

[B47] Lochhead J. J. Davis T. P. (2019). Perivascular and perineural pathways involved in brain delivery and distribution of drugs after intranasal administration. *Pharmaceutics* 11:598. 10.3390/pharmaceutics11110598 31726721 PMC6921024

[B48] Lochhead J. J. Thorne R. G. (2012). Intranasal delivery of biologics to the central nervous system. *Adv. Drug Deliv. Rev*. 64 614–628. 10.1016/j.addr.2011.11.002 22119441

[B49] Long Q. Upadhya D. Hattiangady B. Kim D. K. An S. Y. Shuai B. (2017). Intranasal MSC-derived A1-exosomes ease inflammation, and prevent abnormal neurogenesis and memory dysfunction after status epilepticus. *Proc. Natl. Acad. Sci. U. S. A.* 114 E3536–E3545. 10.1073/pnas.1703920114 28396435 PMC5410779

[B50] Losurdo M. Pedrazzoli M. D’Agostino C. Elia C. A. Massenzio F. Lonati E. (2020). Intranasal delivery of mesenchymal stem cell-derived extracellular vesicles exerts immunomodulatory and neuroprotective effects in a 3xTg model of Alzheimer’s disease. *Stem Cells Transl. Med*. 9 1068–1084. 10.1002/sctm.19-0327 32496649 PMC7445021

[B51] Lu M. H. Ji W. L. Chen H. Sun Y. Y. Zhao X. Y. Wang F. (2021). Intranasal transplantation of human neural stem cells ameliorates Alzheimer’s disease-like pathology in a mouse model. *Front. Aging Neurosci*. 13:650103. 10.3389/fnagi.2021.650103 33776747 PMC7987677

[B52] Marsh S. E. Yeung S. T. Torres M. Lau L. Davis J. L. Monuki E. S. (2017). HuCNS-SC human NSCs fail to differentiate, form ectopic clusters, and provide no cognitive benefits in a transgenic model of Alzheimer’s disease. *Stem Cell Rep.* 8 235–248. 10.1016/j.stemcr.2016.12.019 28199828 PMC5312253

[B53] Martin P. Wagh V. Reis S. A. Erdin S. Beauchamp R. L. Shaikh G. (2020). TSC patient-derived isogenic neural progenitor cells reveal altered early neurodevelopmental phenotypes and rapamycin-induced MNK-eIF4E signaling. *Mol. Autism* 11:2. 10.1186/s13229-019-0311-3 31921404 PMC6945400

[B54] McGinley L. M. Kashlan O. N. Bruno E. S. Chen K. S. Hayes J. M. Kashlan S. R. (2018). Human neural stem cell transplantation improves cognition in a murine model of Alzheimer’s disease. *Sci. Rep*. 8:14776. 10.1038/s41598-018-33017-6 30283042 PMC6170460

[B55] Mulcahy L. A. Pink R. C. Carter D. R. (2014). Routes and mechanisms of extracellular vesicle uptake. *J. Extracell. Vesicles* 3:24641. 10.3402/jev.v3.24641 25143819 PMC4122821

[B56] Muñoz S. S. Engel M. Balez R. Do-Ha D. Cabral-da-Silva M. C. Hernández D. (2020). A simple differentiation protocol for generation of induced pluripotent stem cell-derived basal forebrain-like cholinergic neurons for Alzheimer’s disease and frontotemporal dementia disease modeling. *Cells* 9:2018. 10.3390/cells9092018 32887382 PMC7564334

[B57] Oakley H. Cole S. L. Logan S. Maus E. Shao P. Craft J. (2006). Intraneuronal beta-amyloid aggregates, neurodegeneration, and neuron loss in transgenic mice with five familial Alzheimer’s disease mutations: Potential factors in amyloid plaque formation. *J. Neurosci*. 26 10129–10140. 10.1523/jneurosci.1202-06.2006 17021169 PMC6674618

[B58] Ogaki A. Ikegaya Y. Koyama R. (2021). Extracellular vesicles taken up by astrocytes. *Int. J. Mol. Sci*. 22:10553. 10.3390/ijms221910553 34638890 PMC8508591

[B59] Osawa S. Kurachi M. Yamamoto H. Yoshimoto Y. Ishizaki Y. (2017). Fibronectin on extracellular vesicles from microvascular endothelial cells is involved in the vesicle uptake into oligodendrocyte precursor cells. *Biochem. Biophys. Res. Commun.* 488 232–238. 10.1016/j.bbrc.2017.05.049 28499870

[B60] Pauwels M. J. Vandendriessche C. Vandenbroucke R. E. (2021). Special delevery: Extracellular vesicles as promising delivery platform to the brain. *Biomedicines* 9:1734. 10.3390/biomedicines9111734 34829963 PMC8615927

[B61] Polanco J. C. Li C. Durisic N. Sullivan R. Götz J. (2018). Exosomes taken up by neurons hijack the endosomal pathway to spread to interconnected neurons. *Acta Neuropathol. Commun*. 6:10. 10.1186/s40478-018-0514-4 29448966 PMC5815204

[B62] Pusic K. M. Pusic A. D. Kraig R. P. (2016). Environmental enrichment stimulates immune cell secretion of exosomes that promote CNS myelination and may regulate inflammation. *Cell Mol. Neurobiol*. 36 313–325. 10.1007/s10571-015-0269-4 26993508 PMC4860060

[B63] Santamaria S. Gagliani M. C. Bellese G. Marconi S. Lechiara A. Dameri M. (2021). Imaging of endocytic trafficking and extracellular vesicles released under neratinib treatment in ERBB2(+) breast cancer cells. *J. Histochem. Cytochem*. 69 461–473. 10.1369/00221554211026297 34126793 PMC8246527

[B64] Shetty A. K. Upadhya R. (2021). Extracellular vesicles in health and disease. *Aging Dis*. 12 1358–1362. 10.14336/ad.2021.0827 34527414 PMC8407881

[B65] Spellicy S. E. Kaiser E. E. Bowler M. M. Jurgielewicz B. J. Webb R. L. West F. D. (2020). Neural stem cell extracellular vesicles disrupt midline shift predictive outcomes in porcine ischemic stroke model. *Transl. Stroke Res*. 11 776–788. 10.1007/s12975-019-00753-4 31811639 PMC7340639

[B66] Sun M. K. Passaro A. P. Latchoumane C. F. Spellicy S. E. Bowler M. Goeden M. (2020). Extracellular vesicles mediate neuroprotection and functional recovery after traumatic brain injury. *J. Neurotrauma* 37 1358–1369. 10.1089/neu.2019.6443 31774030 PMC7249471

[B67] Théry C. Ostrowski M. Segura E. (2009). Membrane vesicles as conveyors of immune responses. *Nat. Rev. Immunol.* 9 581–593. 10.1038/nri2567 19498381

[B68] Tian T. Cao L. He C. Ye Q. Liang R. You W. (2021). Targeted delivery of neural progenitor cell-derived extracellular vesicles for anti-inflammation after cerebral ischemia. *Theranostics* 11 6507–6521. 10.7150/thno.56367 33995671 PMC8120222

[B69] Upadhya D. Hattiangady B. Castro O. W. Shuai B. Kodali M. Attaluri S. (2019). Human induced pluripotent stem cell-derived MGE cell grafting after status epilepticus attenuates chronic epilepsy and comorbidities via synaptic integration. *Proc. Natl. Acad. Sci. U. S. A.* 116 287–296. 10.1073/pnas.1814185115 30559206 PMC6320542

[B70] Upadhya D. Shetty A. K. (2019). Extracellular vesicles as therapeutics for brain injury and disease. *Curr. Pharm. Des*. 25 3500–3505. 10.2174/1381612825666191014164950 31612823

[B71] Upadhya D. Shetty A. K. (2021a). Promise of extracellular vesicles for diagnosis and treatment of epilepsy. *Epilepsy Behav.* 121:106499. 10.1016/j.yebeh.2019.106499 31636006 PMC7165061

[B72] Upadhya R. Madhu L. N. Attaluri S. Gitaí D. L. G. Pinson M. R. Kodali M. (2020a). Extracellular vesicles from human iPSC-derived neural stem cells: miRNA and protein signatures, and anti-inflammatory and neurogenic properties. *J. Extracell. Vesicles* 9:1809064. 10.1080/20013078.2020.1809064 32944193 PMC7480597

[B73] Upadhya R. Zingg W. Shetty S. Shetty A. K. (2020b). Astrocyte-derived extracellular vesicles: Neuroreparative properties and role in the pathogenesis of neurodegenerative disorders. *J. Control Release* 323 225–239. 10.1016/j.jconrel.2020.04.017 32289328 PMC7299747

[B74] Upadhya R. Madhu L. N. Rao S. Shetty A. K. (2022). Proficiency of extracellular vesicles from hiPSC-derived neural stem cells in modulating proinflammatory human microglia: Role of pentraxin-3 and miRNA-21-5p. *Front. Mol. Neurosci*. 15:845542. 10.3389/fnmol.2022.845542 35656007 PMC9152457

[B75] Upadhya R. Shetty A. K. (2021b). Extracellular vesicles for the diagnosis and treatment of Parkinson’s disease. *Aging Dis*. 12 1438–1450. 10.14336/ad.2021.0516 34527420 PMC8407884

[B76] Veitch D. P. Weiner M. W. Aisen P. S. Beckett L. A. DeCarli C. Green R. C. (2022). Using the Alzheimer’s Disease Neuroimaging Initiative to improve early detection, diagnosis, and treatment of Alzheimer’s disease. *Alzheimers Dement*. 18 824–857. 10.1002/alz.12422 34581485 PMC9158456

[B77] Verkhratsky A. Matteoli M. Parpura V. Mothet J. P. Zorec R. (2016). Astrocytes as secretory cells of the central nervous system: Idiosyncrasies of vesicular secretion. *EMBO J.* 35 239–257. 10.15252/embj.201592705 26758544 PMC4741299

[B78] Vogel A. D. Upadhya R. Shetty A. K. (2018). Neural stem cell derived extracellular vesicles: Attributes and prospects for treating neurodegenerative disorders. *Ebiomedicine* 38 273–282. 10.1016/j.ebiom.2018.11.026 30472088 PMC6306394

[B79] Wang S. Zeng M. Ren Y. Han S. Li J. Cui W. (2021a). In vivo reduction of hippocampal Caveolin-1 by RNA interference alters morphine addiction and neuroplasticity changes in male mice. *Neurosci. Lett*. 1:135742. 10.1016/j.neulet.2021.135742 33607203

[B80] Wang S. Ichinomiya T. Terada Y. Wang D. Patel H. H. Head B. P. (2021b). Synapsin-Promoted caveolin-1 overexpression maintains mitochondrial morphology and function in PSAPP Alzheimer’s disease mice. *Cells* 10:2487. 10.3390/cells10092487 34572135 PMC8467690

[B81] Wang W. Zhao F. Ma X. Perry G. Zhu X. (2020). Mitochondria dysfunction in the pathogenesis of Alzheimer’s disease: Recent advances. *Mol. Neurodegener*. 15:30. 10.1186/s13024-020-00376-6 32471464 PMC7257174

[B82] Wiklander O. P. B. Nordin J. Z. O’Loughlin A. Gustafsson Y. Corso G. Mäger I. (2015). Extracellular vesicle in vivo biodistribution is determined by cell source, route of administration and targeting. *J. Extracell. Vesicles* 4:26316. 10.3402/jev.v4.26316 25899407 PMC4405624

[B83] Willis C. M. Nicaise A. M. Hamel R. Pappa V. Peruzzotti-Jametti L. Pluchino S. (2020a). Harnessing the Neural Stem Cell Secretome for Regenerative Neuroimmunology. *Front. Cell. Neurosci*. 14:590960. 10.3389/fncel.2020.590960 33250716 PMC7674923

[B84] Willis C. M. Nicaise A. M. Peruzzotti-Jametti L. Pluchino S. (2020b). The neural stem cell secretome and its role in brain repair. *Brain Res.* 1729:146615. 10.1016/j.brainres.2019.146615 31863730

[B85] Xia Y. Hu G. Chen Y. Yuan J. Zhang J. Wang S. (2021). Embryonic stem cell derived small extracellular vesicles modulate regulatory T cells to protect against ischemic stroke. *ACS Nano* 15 7370–7385. 10.1021/acsnano.1c00672 33733738

[B86] Xin H. Li Y. Liu Z. Wang X. Shang X. Cui Y. (2013). MiR-133b promotes neural plasticity and functional recovery after treatment of stroke with multipotent mesenchymal stromal cells in rats via transfer of exosome-enriched extracellular particles. *Stem Cells* 31 2737–2746. 10.1002/stem.1409 23630198 PMC3788061

[B87] Yue C. Feng S. Chen Y. Jing N. (2022). The therapeutic prospects and challenges of human neural stem cells for the treatment of Alzheimer’s Disease. *Cell Regen*. 11:28. 10.1186/s13619-022-00128-5 36050613 PMC9437172

[B88] Yue K. Y. Zhang P. R. Zheng M. H. Cao X. L. Cao Y. Zhang Y. Z. (2019). Neurons can upregulate Cav-1 to increase intake of endothelial cells-derived extracellular vesicles that attenuate apoptosis via miR-1290. *Cell Death Dis*. 10:869. 10.1038/s41419-019-2100-5 31740664 PMC6861259

[B89] Zakharov A. Papaiconomou C. Johnston M. (2004). Lymphatic vessels gain access to cerebrospinal fluid through unique association with olfactory nerves. *Lymphat. Res. Biol.* 2 139–146. 10.1089/lrb.2004.2.139 15609813

[B90] Zhang T. Ke W. Zhou X. Qian Y. Feng S. Wang R. (2019). Human neural stem cells reinforce hippocampal synaptic network and rescue cognitive deficits in a mouse model of Alzheimer’s disease. *Stem Cell Rep.* 13 1022–1037. 10.1016/j.stemcr.2019.10.012 31761676 PMC6915849

